# Genome‐wide analysis of odorant‐binding proteins and chemosensory proteins in the sweet potato whitefly, *Bemisia tabaci*

**DOI:** 10.1111/1744-7917.12576

**Published:** 2018-03-26

**Authors:** Yang Zeng, Yu‐Ting Yang, Qing‐Jun Wu, Shao‐Li Wang, Wen Xie, You‐Jun Zhang

**Affiliations:** ^1^ Department of Plant Protection, Institute of Vegetables and Flowers Chinese Academy of Agricultural Sciences Beijing China; ^2^ Department of Agriculture of Yangtze University Jingzhou Hubei Province China

**Keywords:** *Bemisia tabaci*, CSPs, expression patterns, genome‐wide identification, OBPs, phylogenetic

## Abstract

Odorant‐binding proteins (OBPs) and chemosensory proteins (CSPs) of insects are thought to play roles in olfactory recognition affecting host choice, copulation, reproduction and other behaviors. Previous descriptions of OBPs and CSPs in the whitefly *Bemisia tabaci* often provided no or incomplete genetic information. In this study, we present a genome‐wide and transcriptome‐wide investigation of the OBPs and CSPs in *B. tabaci* MEAM1 (Middle East‐Asia Minor1 species). Eight OBP and 19 CSP genes were identified that covered all previous sequences. Phylogenetic analyses showed that the CSP genes had a lineage‐specific expansion (*BtabBCSP1*, *BtabBCSP3*, *BtabBCSP13*, *BtabBCSP17*, *BtabBCSP18* and *BtabBCSP19*). Expression profiling of OBPs and CSPs by transcriptome sequencing and quantitative real‐time polymerase chain reaction (qPCR) revealed that expression patterns differed among developmental stages of *B. tabaci* MEAM1. Five OBP genes and 11 CSP genes significantly differed between males and females; four of the 19 CSP genes were highly expressed in adults, while two were highly expressed in nymphs. The expression profiles of the OBP and CSP genes in different tissues of *B. tabaci* MEAM1 adults were analyzed by qPCR. Four OBP genes found in *B. tabaci* MEAM1 were highly expressed in the head. Conversely, only two CSPs were enriched in the head, while the other six CSPs were specifically expressed in other tissues. Our results provide a foundation for future research on OBPs and CSPs in *B. tabaci*.

## Introduction

The olfactory recognition system plays a critical role in feeding, mating, oviposition, and other important behaviors of insects. The first step in olfactory recognition is the solubilization and transport of odor molecules from the external environment to the olfactory sensory neurons. In insects, this task is performed by two major families of small, soluble proteins: odorant‐binding proteins (OBPs) and chemosensory proteins (CSPs) (Vogt & Riddiford, 1981; Vogt *et al*., 1991; Angeli *et al*., 1999; Pelosi *et al*., 2006, 2014, 2018). OBPs provide the initial molecular interactions with chemical signals such as pheromones and host odors and are thought to ferry the semiochemical molecules across the antennal sensillum lymph to the olfactory receptors (Steinbrecht, 1998). OBPs are small (10–30 kDa), globular and abundant water‐soluble acidic proteins with a pattern of six conserved cysteine residues. These cysteine residues are paired into three interlocked disulfide bridges, which together with other amino acids form an odorant‐binding pocket that binds and protects small hydrophobic ligands (Leal *et al*., 1999; Scaloni *et al*., 1999; Laughlin *et al*., 2008). OBPs in insects have rich and expanded roles in pheromone signal transduction (Xu *et al*., 2005) and in the manipulation of host selection and mating behavior (Hooper *et al*., 2009; Pannure *et al*., 2012), and are also important for the identification of cryptic species (Lardeux *et al*., 2012; Gholizadeh *et al*., 2015). CSPs are small, soluble proteins that are abundant in the sensilla lymph and that have many functions (Wanner *et al*., 2004; Gong *et al*., 2007). Like OBPs, CSPs are odor‐binding proteins (Ban *et al*., 2003; Ozaki *et al*., 2005; Foret *et al*., 2007; Zhou *et al*., 2010; Li *et al*., 2013), but CSPs have fewer conserved cysteine residues and more conserved nucleotide sequences than OBPs across insect species (Pelosi *et al*., 2005). CSPs have more functions than OBPs in non‐sensory organs of insects, and these functions include pheromone delivery, solubilization of nutrients, and the development of insecticide resistance (Kamikouchi *et al*., 2004; Wanner *et al*., 2005; Maleszka *et al*., 2007; Xu *et al*., 2009; Liu *et al*., 2010; Guo *et al*., 2011, 2012; Zhou *et al*., 2013; Zhang *et al*., 2013; Jean‐François, 2014). CSPs can also act as effector proteins to trigger plant physiological defenses (Bos *et al*., 2010).

The sibling and cryptic species of the whitefly *Bemisia tabaci* include some of the world's most damaging agricultural pests and are considered among the World's Worst Invasive Species (Global Invasive Species Database: http://www.issg.org/database/welcome/). Among the *B. tabaci* sibling species, *Bemisia* Middle East‐Asia Minor 1 (MEAM1 or ‘B’) and *Bemisia* Mediterranean (MED or ‘Q’) are the most extensively studied. They are considered to be highly invasive and destructive pests in many parts of the world because of their broad host range and their ability to transmit viral pathogens of plants (Jones, 2003; De Barro *et al*., 2011; Gilbertson *et al*., 2015; Wan & Yang, 2016).

To date, OBPs and CSPs and the genes that encode them have been partly identified in *B. tabaci* based on expressed sequence tags and head transcriptome data (Li *et al*., 2012, 2014, 2016; Wang *et al*., 2016, 2017). However, the sequences obtained are incomplete. A comprehensive understanding of OBPs and CSPs in *B. tabaci* requires a more complete genome‐wide analysis. In this research, we used previously published data on *B. tabaci* genomes (Chen *et al*., 2016; Xie *et al*., 2017) and new antenna transcriptome data (obtained in the current study) to complete a genome‐wide analysis of OBP and CSP gene families in *B. tabaci* MEAM1. We systematically classified, characterized, and phylogenetically analyzed the OBP and CSP genes. By searching both genomic and transcriptomic data and experimentally validating the results, we identified eight candidate OBP genes and 19 candidate CSP genes in this species. We also used RPKM (reads per kilobase per million mapped reads) and quantitative real‐time polymerase chain reaction (qPCR) to determine the expression profiles of these genes in different developmental stages and different tissues. In addition to providing a framework for further research on *B. tabaci* OBPs and CSPs, the results will be useful for comparing OBP and CSP genes and proteins among different species.

## Materials and methods

### Insect rearing and sample preparation

A *B. tabaci* MEAM1 population was maintained on cotton plants at 27 ± 1 °C with an L : D 16 : 8 photoperiod and a relative humidity (RH) of 70% ± 10%. Every three to five generations, the purity of the strain was monitored using PCR and the sequence of mitochondrial cytochrome oxidase I (*mtCO I*) gene (Chu *et al*., 2010). Samples of stages (eggs, the four nymph stages, females, males) and tissues (head, abdomen and mixture of thorax, legs and wings) were separately collected from the *B. tabaci* MEAM1 population, rapidly frozen in liquid nitrogen, and stored at −80 °C.

### RNA isolation, cDNA library construction, Illumina sequencing and antennae transcriptome assembly

RNA from the anatomical antennae tissues of thousands of adults of MEAM1 was extracted with Trizol reagent (Invitrogen, Carlsbad, CA, USA). according to the manufacturer's instructions, and RNA purity and degradation were checked on 1% agarose gels. RNA integrity was further confirmed using the 2100 Bioanalyzer (Agilent Technologies, Santa Clara, CA, USA) with a minimum RNA integrity number of 8. Poly (A)‐containing RNA was separated from the total RNA using the Dynabeads® mRNA purification kit (Invitrogen, Carlsbad, CA, USA), and the quality was verified on a denaturing gel. The messenger RNA (mRNA) was then used for SMARTer first‐strand complementary DNA (cDNA) synthesis using the SMARTer Ultra Low Input RNA for Illumina Sequencing Components – HV (cat. nos. 634822, 634825, 634827 and 634831). This was followed by full‐length double‐stranded cDNA (ds‐cDNA) amplification using limiting dilution PCR. PCR‐amplified cDNA was purified by using SPRI Ampure Beads, and purity was confirmed by using the Agilent 2100 BioAnalyzer. After covaris shearing of full‐length cDNA, the Low Input Library Prep Kit (cat. no. 634947) was used to create the final cDNA library. The paired‐end cDNA libraries (200 bp size) were prepared following the manufacturer's recommendations and sequenced on an Illumina GAII platform. The resulting high‐quality cleaned reads were assembled *de novo* into contigs using Trinity (trinityrnaseq_r20131110) with default parameters except that ‘min_kmer_cov’ was set to 2 (Friedman *et al*., 2011).

### Identification of putative OBPs and CSPs in B. tabaci

The computational pipeline is detailed in Figure S3. The protein sequences of known OBPs and CSPs were used to search the *B. tabaci* (MEAM1) genome 1.0, the *B. tabaci* (MEAM1) genome (Chen *et al*., 2016), and the antenna transcriptome using the program TBLASTN with an e‐value threshold of 10^−5^. The sequences meeting the criteria were collected as candidate OBP/CSP sequences. After removal of the identical sequences, the remaining sequences were classified into two types (OBPs and CSPs). Putative OBP/CSP sequences were confirmed by subjecting them to BLASTX analysis with the non‐redundant protein sequence (NR) at GenBank (http://www.ncbi.nlm.nih.gov/). The conserved domains of these identified OBPs and CSPs were predicted using SMART (simple modular architecture research tool, http://smart.emblheidelberg.de/) (Letunic *et al*., 2015) and were confirmed using the National Center for Biotechnology Information conserved domain search service tool. All candidate OBP and CSP sequences were further validated by cloning and sequencing. Gene‐specific primers were designed and used to clone the open reading frame (ORF) or partial sequences of each OBP and CSP. The method of identification of putative OBPs and CSPs in *B. tabaci* MED is the same as above in *B. tabaci* MEAM1 except for MEAM1 antenna transcriptome application.

### Sequence and phylogenetic analysis

The putative N‐terminal signal peptides and the most likely cleavage sites were predicted using the SignalP V4.1 program (http://www.cbs.dtu.dk/services/SignalP/). Sequences were aligned using the program ClustalW with default gap penalty parameters of gap opening 10 and extension 0.2. A neighbor‐joining tree was constructed using the program MEGA 6.0 with a p‐distance model and a pairwise deletion of gaps (Tamura *et al*., 2013). The bootstrap support of tree branches was assessed by re‐sampling amino acid positions 1000 times. Phylogenetic trees were then presented in circular shape and colored taxonomically using online tools provided by Evolview (He *et al*., 2016).

### Motif analysis

A total of 120 of OBPs and 64 CSPs from *B. tabaci* MEAM1, *B. tabaci* MED and other insects (Supplementary file) were used for motif discovery and pattern analysis. The MEME (version 4.12.0) on the line server (http://meme-suite.org/index.html) was used to discover and analyze the motifs in this analysis. The parameters used were as follows: minimum width = 6, maximum width = 10, and the maximum number of motifs to find = 6.

### Expression profiling of OBPs and CSPs

Expression profiles of OBPs and CSPs in different developmental stages of *B. tabaci* MEAM1 were obtained using transcriptome data. Samples were represented by three biological replicates that were independently processed. Total RNA was extracted using Trizol reagent according to the manufacturer's instructions (Invitrogen, Carlsbad, CA, USA). RNA was quantified using a Nanodrop 2000 (Thermo Scientific, Wilmington, DE, USA), and purity was checked on 1% agarose gels. RNA‐seq libraries were constructed as previously described and sequenced on a HiSeq 2500 system according to the manufacturer's instructions with sequencing at 125 bp (PE125, library size is 280–320 bp). The software Fastq_clean was used for RNA‐seq data cleaning and quality control (Zhang *et al*., 2014). The raw RNA‐seq reads were filtered with Fastq_clean software by trimming low‐quality (Q value < 20) nucleotides on both ends, clipping the adapter and barcode sequences from the 3′ end, and discarding the ribosomal RNA (rRNA) sequence. We then aligned the high‐quality cleaned RNA‐seq reads to the pre‐prepared RNA sequence data set with the Bowtie program allowing one mismatch. Following alignments, raw counts for each transcript and in each sample were derived and normalized to RPKM. Statistical analyses and plotting were conducted using the software R v2.15.3 with the Bioconductor packages (Gao *et al*., 2014). Differentially expressed genes (fold‐change > 2 and adjusted *P*‐value < 0.05) between two selected conditions were identified with the DESeq package. The transcript levels of *B. tabaci* CSPs and OBPs in different developmental stages were determined by calculating log2 (RPKM + 1) values.

Besides transcriptomic validation, qPCR analysis was used to confirm mRNA expression of CSPs in *B. tabaci*. Based on the RPKM value generated from the RNAseq data, we selected nine genes (differentially expressed genes between adult and egg, fold‐change > 2 and adjusted *P*‐value < 0.01) that are representative of all of the *B. tabaci* CSPs for the qPCR validation study. We also selected eight CSP genes and four OBP genes (differentially expressed genes between males and females, fold‐change > 2 and adjusted *P*‐value < 0.01) to confirm the transcript levels in different tissues. qPCR was conducted using an ABI PRISM 7500 Real‐time PCR System (Applied Biosystems, Foster City, CA, USA), and non‐treated *B. tabaci* adults were subjected to the analysis. All qPCR analyses included three technical replicates for each of three biological replicates. *EF‐1α* and *SDHA* were selected as the reference genes. The qPCR was carried out in a 20 mL reaction volume containing 10 *μ*L of 2 × Super Real PreMix Plus, 0.4 *μ*L of 50 × ROX Reference Dye, 0.5 *μ*L of forward primer (10 *μ*mol/L), 0.5 *μ*L of reverse primer (10 *μ*mol/L), 1.0 *μ*L of cDNA (300 ng/*μ*L) and 7.6 *μ*L of ribonuclease‐free ddH_2_O. The instructions of the Super Real PreMix Plus (SYBR Green) kit (Tiangen, Beijing, China) were followed. The thermal cycling conditions were polymerase activation at 95°C for 15 min, followed by 40 cycles of denaturation at 95°C for 10 s, annealing at 60°C for 30 s and elongation at 72°C for 32 s. The amplification efficiency was estimated using the following equation: E = [10^ (−1/slope) −1] × 100%, in which the slope was derived by plotting the cycle threshold (Ct) value against six serially diluted template concentrations. The transcript levels of CSP and OBP genes were quantified according to the 2^−ΔΔ^ ${}^{C_{t}}$ method. SPSS 20.0 was used to analyze correlations between qPCR data and RNA‐seq data.

## Results

### Candidate odorant‐binding proteins and phylogenetic analyses in B. tabaci MEAM1

**Figure 1:**
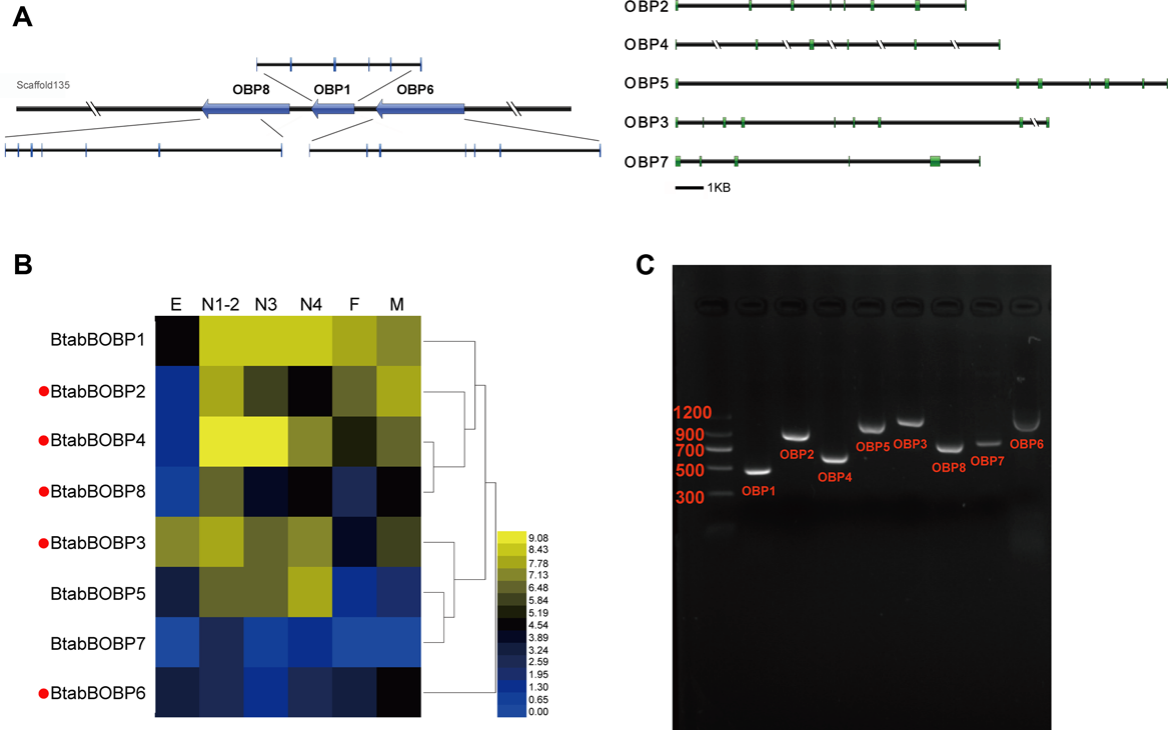
Structure and expression profiles of odorant‐binding protein (OBP) genes in *Bemisia tabaci*. (A) Structures and locations of OBP genes on scaffolds. The blue arrows indicate the transcription orientations of OBP genes on the scaffold. The transcript sequences of OBPs were matched to *B. tabaci* genomic sequences in order to identify the exons and introns. The exon regions are shown with blue boxes. (B) Expression profiles of OBPs in different developmental stages (E = egg, N = nymph stages 1 to 4 as indicated, F = adult female, and M = adult male). The transcript levels were determined by calculating log2 (reads per kilobase per million mapped reads + 1) values. Relative expression levels are indicated by a 15‐grade color scale. ● indicates differentially expressed genes (fold‐change > 2 and adjusted *P*‐value < 0.05) between males and females. (C) Electrophoretic separation of BtabOBPs on an agarose gel.

**Figure 2:**
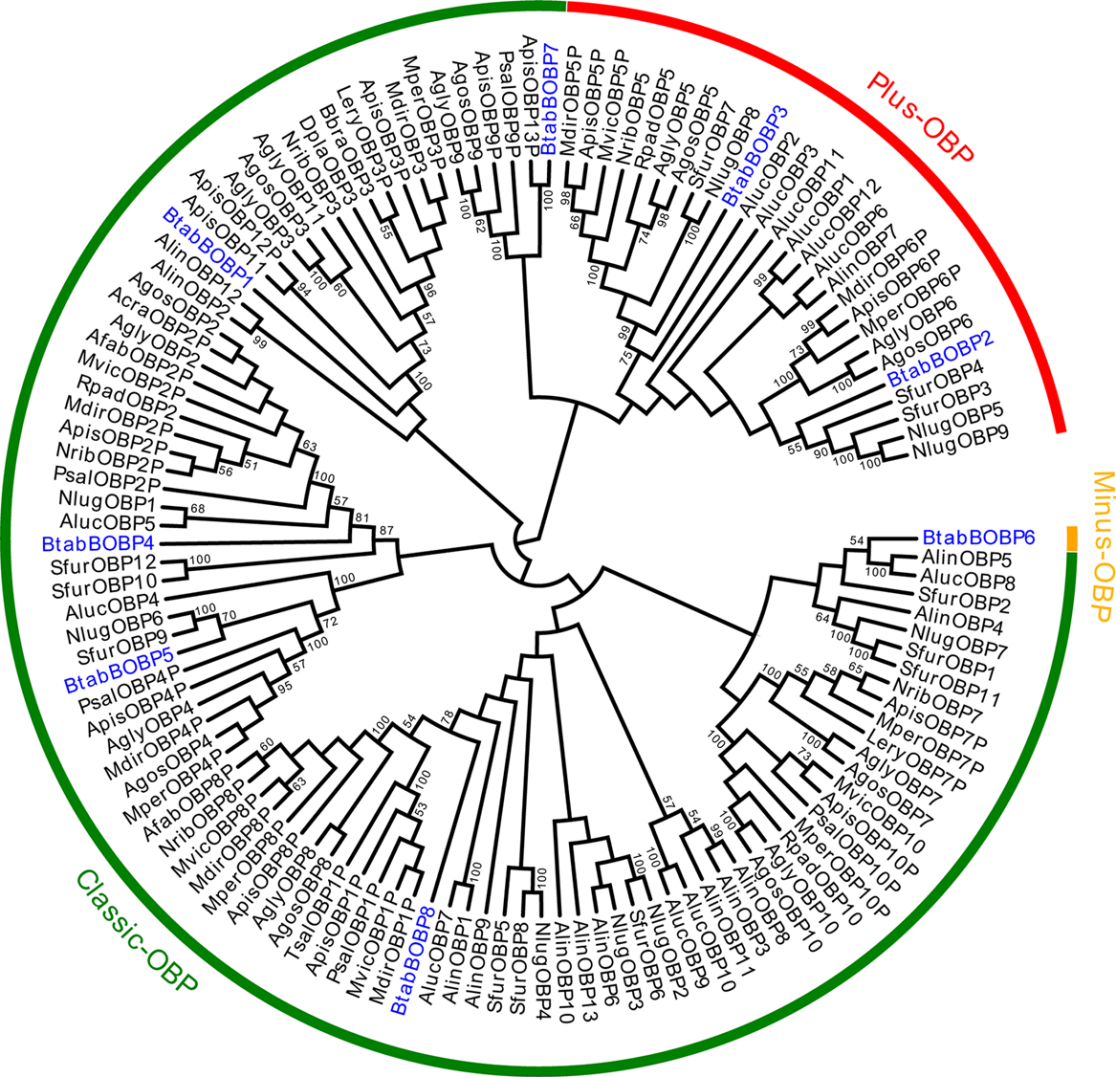
Phylogenetic analysis of the amino acid sequences of BtabBOBPs (indicated by blue) in the context of various hemipteran odorant‐binding proteins (OBPs). Neighbor‐joining tree of the odorant‐binding proteins (OBPs) based on amino acid sequences of *Bemisia tabaci* and other hemipterans. Bootstrap values were calculated with 1000 replications, and those larger than 50% are marked on the nodes. The protein names and sequences of the 112 OBPs used in this analysis are listed in a supplementary file. Btab = *Bemisia tabaci*, Apis = *Acyrthosiphon pisum*, Mper = *Myzus persicae*, Agos = *Aphis gossypii*, Sfur = *Sogatella furcifera*, Nlug = *Nilaparvata lugens*, Psal = *Pterocomma salicis*, Agly = *Aphis glycines*, Aluc = *Apolygus lucorum*, Alin = *Adelphocoris lineolatus*, Rpad = *Rhopalosiphum padi*, Mdir = *Metopolophium dirhodum*, Mvic = *Megoura viciae*, Bbra = *Brevicoryne brassicae*, Lery = *Lipaphis erysimi*, Afab = *Aphis fabae*, Acra = *Aphis craccivora*, Tsal = *Tuberolachnus salignus*, Dpla = *Drepanosiphum platanoidis* and Nrib = *Nasonovia ribis‐nigri*.

Among the eight candidate OBP genes that we identified in the *B. tabaci* genome, three (*BtabBOBP1*, *BtabBOBP6* and *BtabBOBP8*) are located on the same scaffold and have the same orientation (Table 1; Fig. 1). Each of these three OBPs contains 6–7 exons within the 30 kb genomic region. By aligning the sequences and counting the cysteine motifs, we found that *BtabBOBP6* lacks two cysteine residues (C2 and C5) and that *BtabBOBP2* and *BtabBOBP3* have two additional cysteine residues, that is, one after C4 and one after C6. These additional cysteine residues and a conserved proline residue are the key feature of Plus‐C (Fig. S1). We used all of the putative OBPs from *B. tabaci* representative homologous sequences from 20 hemipteran species to build a neighbor‐joining phylogenetic tree. The tree showed a clear cluster representing the Minus‐C OBP class, consisting of *BtabBOBP6* and other similar genes, and a Plus‐C clade OBP class covering *BtabBOBP2* and *BtabBOBP3* (Fig. 2). Remaining OBPs (*BtabBOBP1*, *BtabBOBP4*, *BtabBOBP5*, *BtabBOBP7* and *BtabBOBP8*) were grouped and belonged to the classic clade according to their percentage of similarity among hemipteran species (Fig. 2).

**Table 1:** List of genes encoding odorant‐binding proteins (OBPs) in *Bemisia tabaci* genome

			Location		
Gene name	ORF (bp)	Signal peptide (aa)	Orientation	Start	End
BtabBOBP1	426	1‐24	scaffold_135−	465930	459692
BtabBOBP2	741	1‐22	Scaffold_24−	1533820	1523195
BtabBOBP3	747	1‐26	Scaffold_7−	1994480	1961838
BtabBOBP4	426	1‐19	Scaffold_267−	547353	508414
BtabBOBP5	633	1‐24	Scaffold_277+	384969	402084
BtabBOBP6	435	1‐25	Scaffold_135−	485754	472890
BtabBOBP7†	267	ND	Scaffold_188−	215970	204845
BtabBOBP8	477	1‐21	Scaffold_135−	454393	438508

^†^Indicates that the gene is partial and lacks an intact open reading frame (ORF). ND indicates not detected.

### Expression profiles of the B. tabaci MEAM1 OBPs across developmental stages

Expression significantly differed between males and females for five OBP genes (*BtabBOBP2*, *BtabBOBP3*, *BtabBOBP4*, *BtabBOBP6* and *BtabBOBP8*) (Fig. 1B). Among all developmental stages, expression was highest for *BtabBOBP1* and *BtabBOBP3* and lowest for *BtabBOBP6* and *BtabBOBP7*. Expression of *BtabBOBP2* and *BtabBOBP4* was low in eggs but high in other stages. Expression of *BtabBOBP5* was highest in nymphs followed by eggs.

### mRNA expression of selected B. tabaci MEAM1 OBPs as determined by qPCR across different tissues

**Figure 3:**
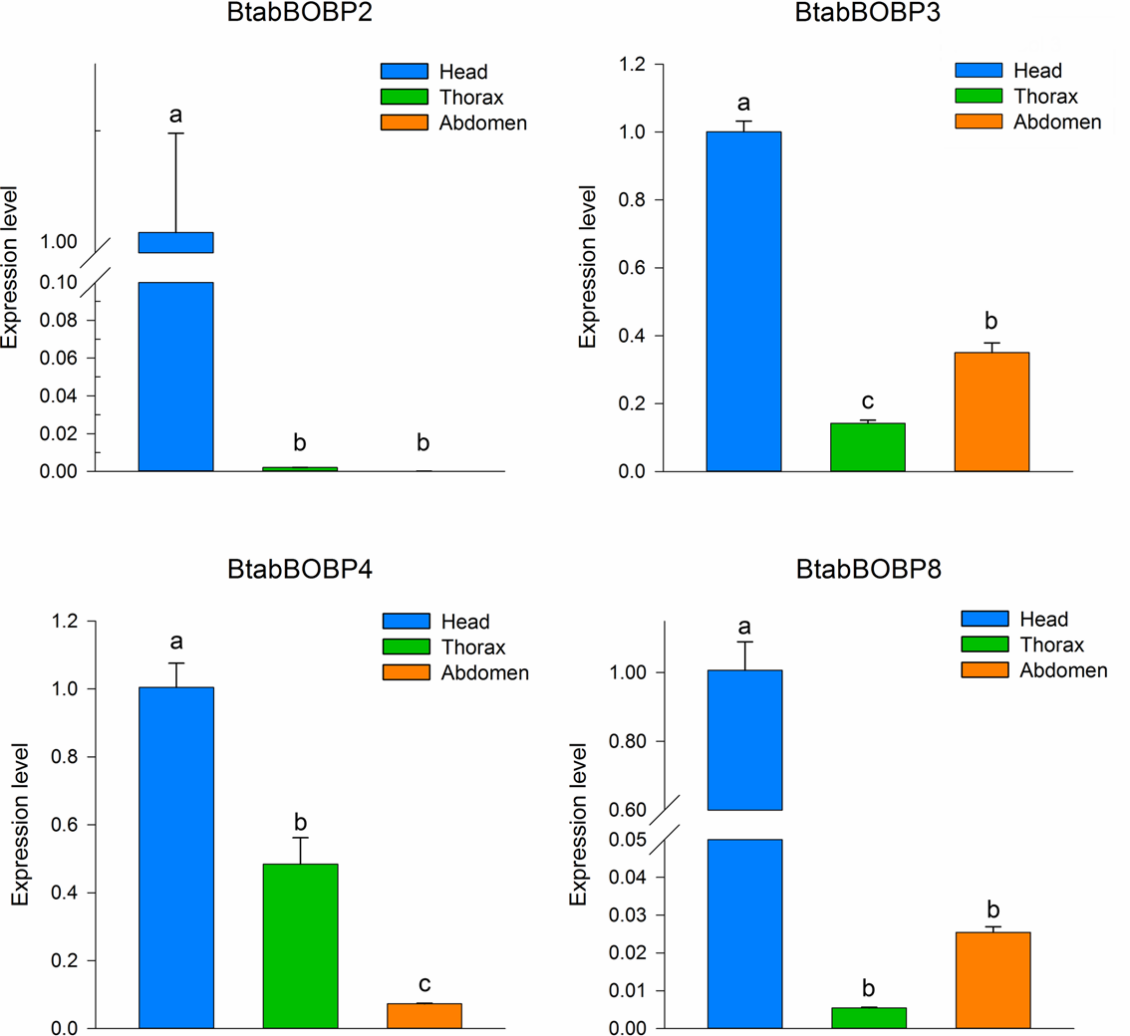
*Bemisia tabaci* odorant‐binding proteins (OBPs) transcript levels in different tissues as measured by quantitative real‐time polymerase chain reaction (qPCR). The expression levels were estimated using 2^−Δ Δ^ ${}^{C_{t}}$ method. Standard error for each sample is represented by error bar and the different letters (a, b, c) above each bar denote significant differences (*P* < 0.05).

qPCR analyses were conducted to measure the expression levels of the four BtabBOBP genes in the head, thorax (mixture of thorax, legs and wings) and abdomen. The results indicated that four genes (*BtabBOBP2*, *BtabBOBP3*, *BtabBOBP4* and *BtabBOBP8*) were approximately 2–100 times more expressed in head than in the other parts (Fig. 3). Furthermore, the two OBPs (*BtabBOBP3* and *BtabBOBP4*) exhibited an expression level of 2.5 and 6.85 times difference between the thorax and abdomen, respectively (*P* < 0.05).

### Candidate chemosensory proteins and phylogenetic analyses in B. tabaci MEAM1

**Figure 4:**
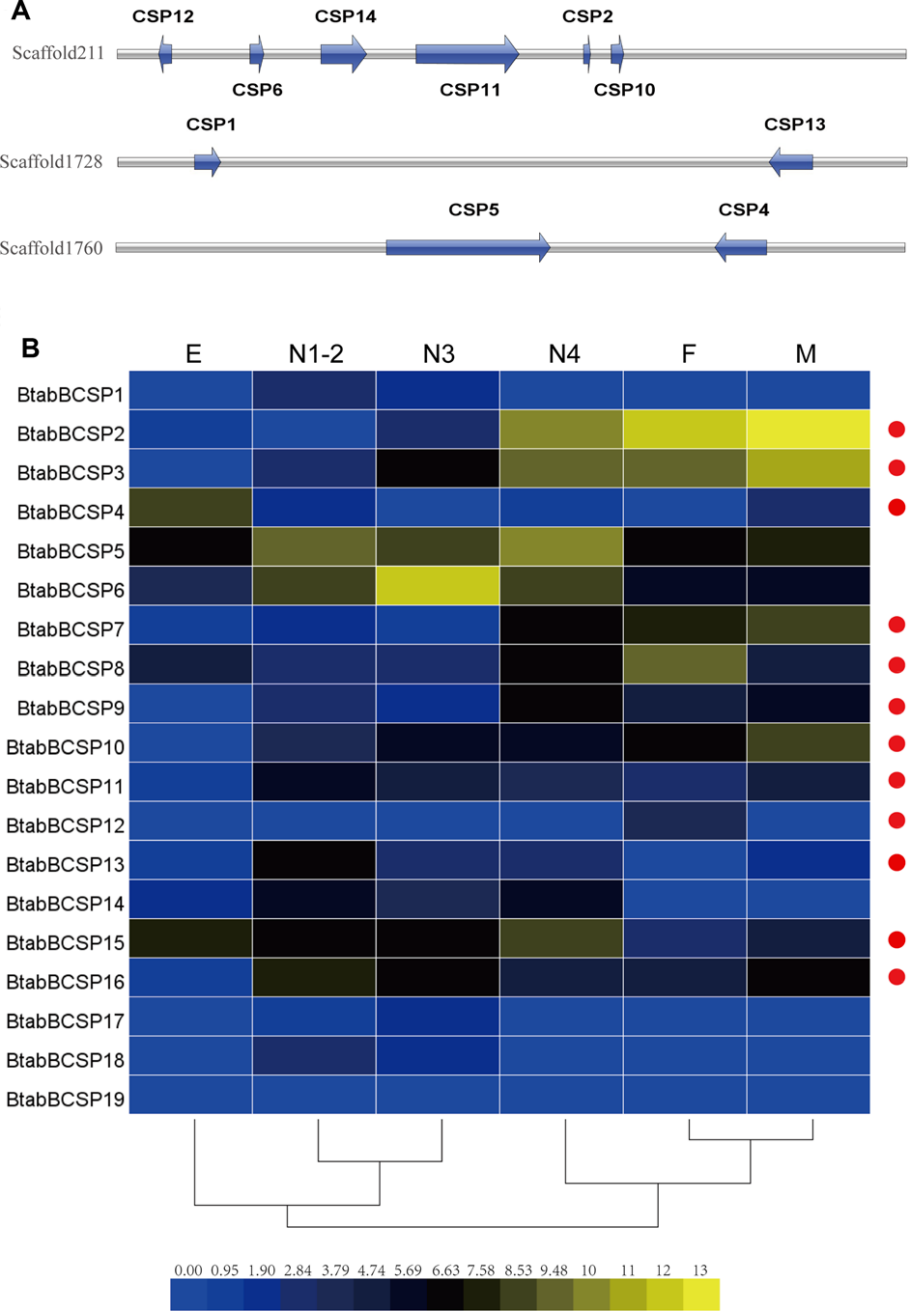
Locations and expression profiles of chemosensory protein (CSP) genes in *Bemisia tabaci*. (A) Locations of CSP genes on the scaffolds. Three gene clusters are located on scaffold211, scaffold1760 and scaffold1728, respectively. Each gene is depicted by arrowheads indicating the orientation of transcription in the scaffold. The exon regions are shown with green boxes. CSPs that could not be aligned well with the scaffold are not shown. (B) Expression profiles of CSPs in different developmental stages. The transcript levels were determined by calculating log2 (reads per kilobase per million mapped reads + 1) values. The levels of expression are indicated by a 15‐grade color scale. ● indicates differentially expressed genes (fold‐change > 2 and adjusted *P*‐value < 0.05) between males and females.

**Figure 5:**
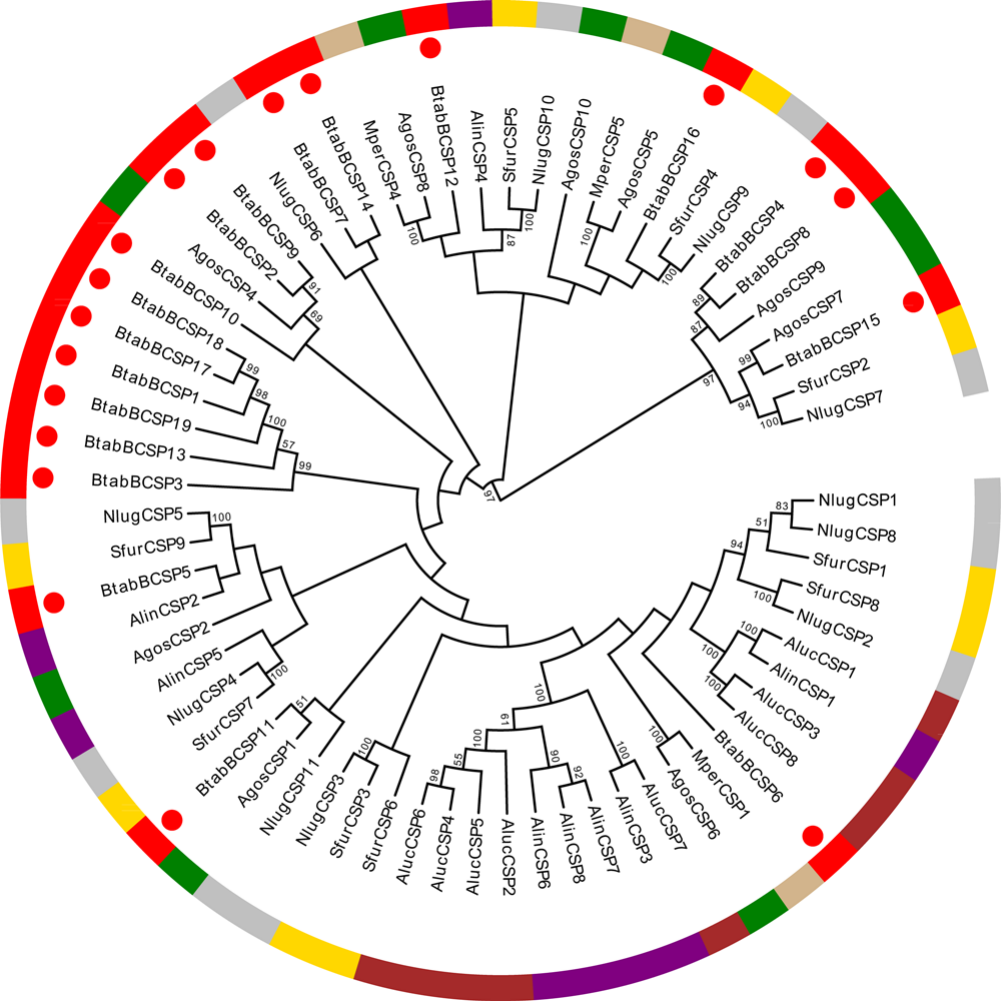
Phylogenetic analysis of the amino acid sequences of BtabBCSPs (indicated by ●) in the context of various hemipteran chemosensory proteins (CSPs). Neighbor‐joining tree of the CSP genes based on amino acid sequences of *Bemisia tabaci* and other insects. Bootstrap values were calculated with 1000 replications, and those larger than 50% are marked on the nodes. The protein names and sequences of the 48 CSPs used in this analysis are in a supplementary file. Alin = *Adelphocoris lineolatus*, Agos = *Aphis gossypii*, Aluc = *Apolygus lucorum*, Mper = *Myzus persicae*, Nlug = *Nilaparvata lugens*, Sfur = *Sogatella furcifera* and Btab = *Bemsia tabaci*.

We identified 19 candidate CSP genes distributed across 11 scaffolds in the *B. tabaci* genome. More than half of them are located within clusters (Table 2). The largest cluster contains six CSP genes, which occur in both orientations on scaffold211 (Fig. 4). The alignment of the predicted *B. tabaci* CSP proteins showed high average pairwise sequence identity between CSP family members. All of the 19 candidate CSPs contain four intact, conserved cysteine residues (Fig. S2). The neighbor‐joining method was used to construct a phylogenetic tree for the CSPs of *B. tabaci* and of seven other hemipteran species (Fig. 5). CSPs in *B. tabaci* are represented in all major clades in phylogenetic trees constructed for these multi‐gene families in hemipterans. Phylogenetic analyses showed that the CSP genes had a lineage‐specific expansion (*BtabBCSP1*, *BtabBCSP3*, *BtabBCSP13*, *BtabBCSP17*, *BtabBCSP18* and *BtabBCSP19*).

**Table 2:** List of genes encoding chemosensory proteins (CSPs) in *Bemisia tabaci* genome

			Location		
Gene name	ORF (bp)	Signal peptide (AA)	Orientation	Start	End
BtabBCSP1	381	1‐19	Scaffold_1728+	4851	6514
BtabBCSP2	393	1‐18	Scaffold_211+	417237	419266
BtabBCSP4	321	1‐19	Scaffold_1760−	41222	37927
BtabBCSP5	483	1‐16	Scaffold_1760+	17122	27511
BtabBCSP6	414	1‐20	Scaffold_211+	319755	323826
BtabBCSP7	384	1‐19	Scaffold_4−	522706	513251
BtabBCSP8	327	1‐20	Scaffold_893−	64906	62662
BtabBCSP9	372	1‐20	Scaffold_14−	2936398	2933040
BtabBCSP10	408	1‐22	Scaffold_211+	425352	428930
BtabBCSP11	738	ND	Scaffold_211+	368338	398455
BtabBCSP12	408	1‐18	Scaffold_211−	296951	293093
BtabBCSP13	381	1‐19	Scaffold_1728−	44016	41267
BtabBCSP14	426	1‐22	Scaffold_211+	340569	353913
BtabBCSP15	336	1‐19	Scaffold_95−	900768	897021
BtabBCSP16	453	1‐16	scaffold74−	114714	108425
BtabBCSP17	381	1‐19	Scaffold_135−	89732	88317
BtabBCSP18	381	1‐19	Scaffold_708+	116559	117889
BtabBCSP19	381	ND	Scaffold_102+	531873	533667

ND indicates not detected. CSPs that could not be aligned well with the scaffold are not shown. ORF, open reading frame.

### Expression profiles of the B. tabaci MEAM1 CSPs across developmental stages

Of the 19 identified CSP genes, the expression of the following 11 significantly differed between females and males of *B. tabaci*: *BtabBCSP2*, *BtabBCSP3*, *BtabBCSP4*, *BtabBCSP7*, *BtabBCSP8*, *BtabBCSP9*, *BtabBCSP10*, *BtabBCSP11*, *BtabBCSP13*, *BtabBCSP15* and *BtabBCSP16*) (Fig. 4B). Expression in adults was highest for *BtabBCSP2*, while expression in eggs was highest for *BtabBCSP4*. The latter gene had the lowest expression in females among the 19 genes. Expression of *BtabBCSP11*, *BtabBCSP13* and *BtabBCSP16* was highest in the 1st and 2nd instar nymphs.

### mRNA expression of selected B. tabaci MEAM1 CSPs as determined by qPCR

**Figure 6:**
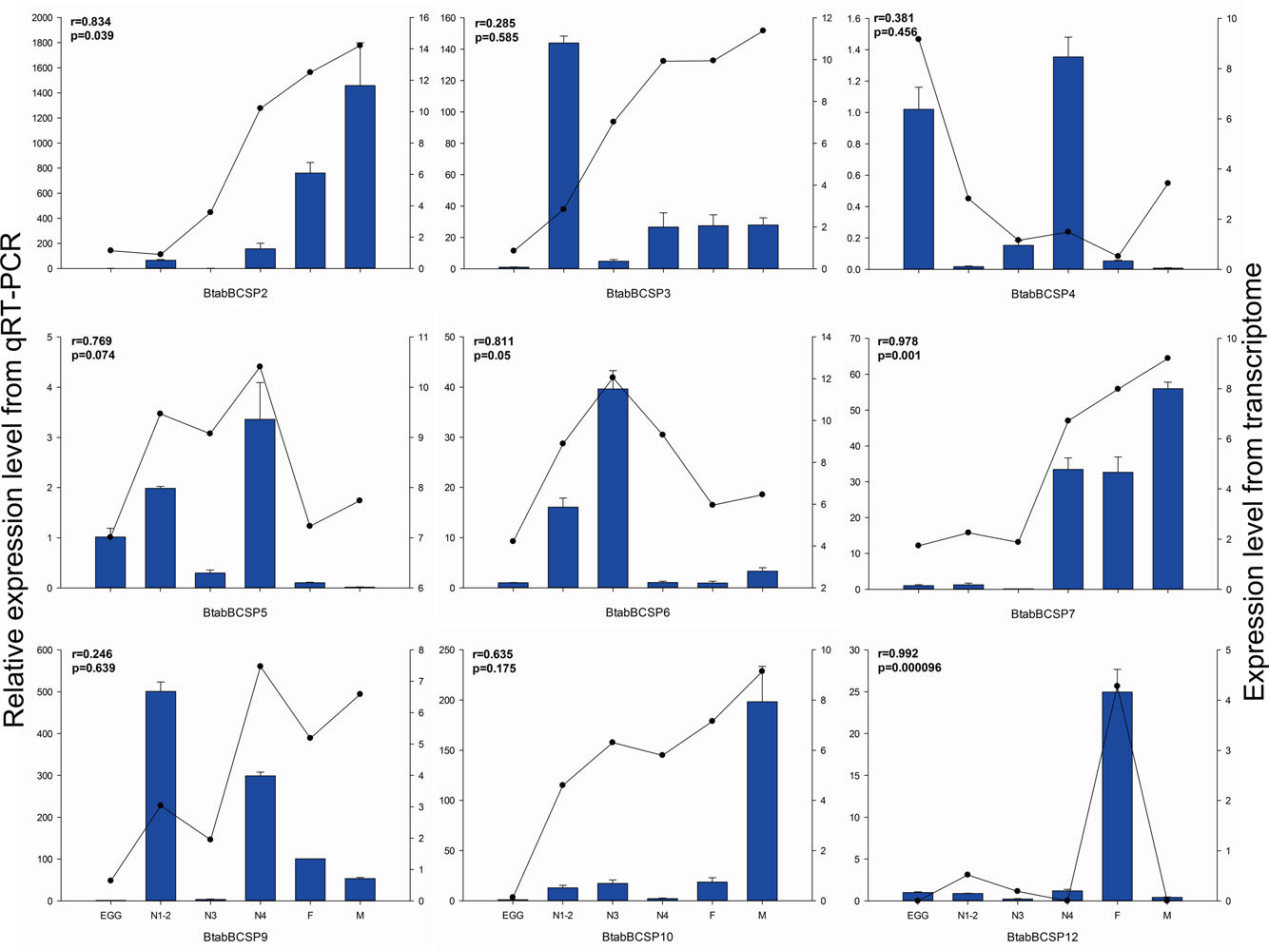
Quantitative real‐time polymerase chain reaction (qPCR)‐based expression profiling of nine selected chemosensory protein (CSP) genes in stages of *Bemisia tabaci*. Total RNA extracted from individual eggs, nymphs and adults of *B. tabaci* were used for the expression analysis of CSP genes by qPCR (dark blue bars) and RNA‐seq (black lines). The relative expression level of each CSP in each developmental stage was normalized to the reference gene *SDHA*. The transcript levels were determined by calculating log2 (reads per kilobase per million mapped reads + 1) values. E = egg, N = nymph stages 1 to 4 as indicated, F = adult female, and M = adult male.

The transcriptome expression profiles of nine selected CSP genes were confirmed by qPCR (Fig. 6). The transcriptome and qPCR expression profiles were highly consistent (*P* ≤ 0.05) for four of the nine CSPs (*BtabBCSP2*, *BtabBCSP6*, *BtabBCSP7* and *BtabBCSP12*). The two kinds of expression profiles also tended to be similar for the other five CSP genes (Fig. 6).

**Figure 7:**
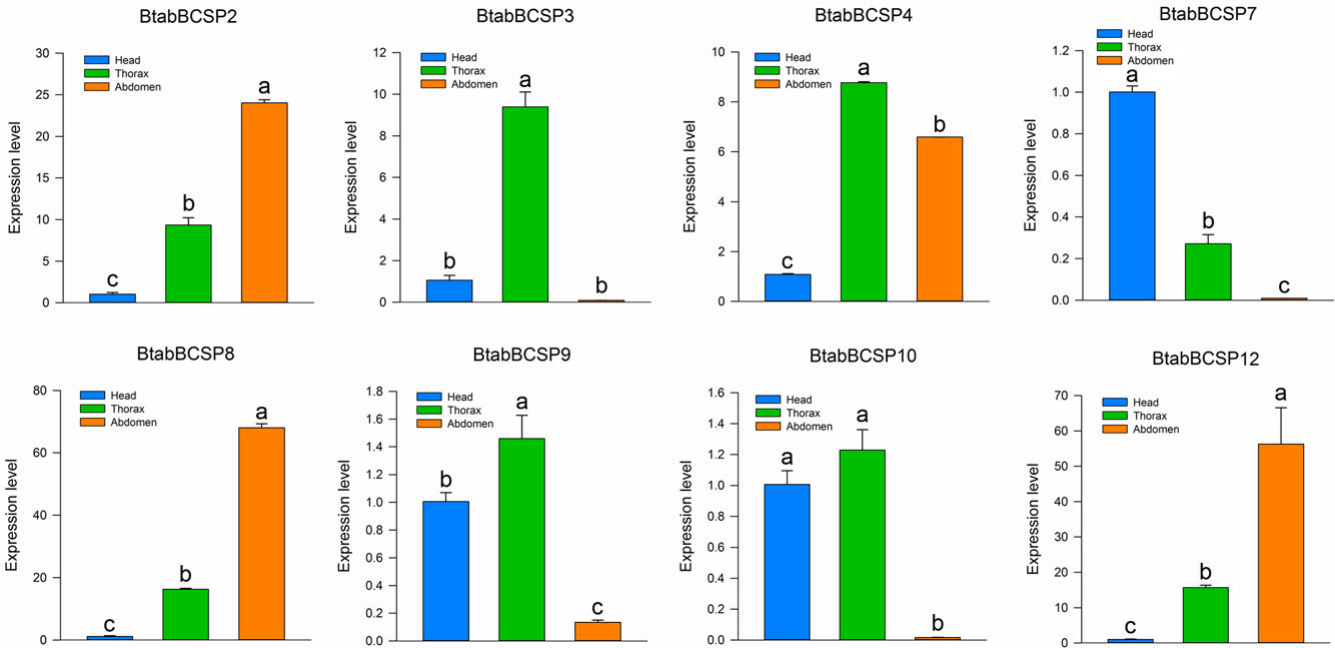
*Bemisia tabaci* chemosensory protein (CSP) transcript levels in different tissues as measured by quantitative real‐time polymerase chain reaction (qPCR). The expression levels were estimated using 2^−Δ Δ^ ${}^{C_{t}}$ method. Standard error for each sample is represented by error bar and the different letters (a, b, c) above each bar denote significant differences (*P* < 0.05).

qPCR analyses were conducted to measure the expression levels of the eight BtabBCSP genes in the head, thorax (mixture of thorax, legs and wings) and abdomen. The results showed that expression level of three genes (*BtabBCSP2*, *BtabBCSP8* and *BtabBCSP12*) were significantly higher in abdomen than that in head and thorax (Fig. 7). Expression in thorax was highest for *BtabBCSP3*, *BtabBCSP4*, *BtabBCSP9* and *BtabBCSP10*, while expression in head was highest for *BtabBCSP7* (Fig. 7).

### Comparison of OBPs and CSPs between B. tabaci MEAM1 and MED

*B. tabaci* MEAM1 and MED both had eight OBP and 19 CSP genes, respectively. Full‐length amino acid sequences alignment showed the sequences of four OBPs and 11 CSPs of *B. tabaci* MEAM1 were completely the same as *B. tabaci* MED but different in *OBP1*, *OBP4*, *OBP5*, *OBP8*, *CSP4*, *CSP5*, *CSP7*, *CSP10*, *CSP11*, *CSP13*, *CSP16* and *CSP17* (Figs. S4 and S5).

## Discussion

Before the current study, the cDNA sequences of OBPs and CSPs had been only partly identified in *B. tabaci* (only eight OBP and 13 CSP sequences had been identified in MED, and only three CSP sequences had been identified in MEAM1), and especially in many cases, the sequences were incomplete (Li *et al*., 2012; Liu *et al*., 2014, 2016; Wang *et al*., 2016a, 2016b, 2017). In the current study, we obtained complete sequences for the eight OBPs and 19 CSPs in MEAM1 (Table 1; Table 2; Table S3) based on genome and antennae transcriptome data sets; these covered (and in some cases completed) all previously published sequences, and added six CSP sequences. We also validated all of these OBP and CSP sequences by molecular cloning and sequencing. It follows that OBP and CSP sequences in this study are accurate and complete. In addition, genome‐wide (no antenna transcriptome) investigation of the OBPs and CSPs in another whitefly *B. tabaci* MED as the same method of MEAM1 was conducted. Sequences comparison between MEAM1 and MED indicated, as expected, that no evident difference were shown regardless of putative sequence number or sequences similarity (Fig. S4 and Fig. S5). This means the two invasive *B. tabaci* MEAM1 and MED may have similar evolution regarding olfactory recognition. These data substantially expand our knowledge of olfactory‐related genes in *B. tabaci* and will be useful for future research concerning the function of these genes and olfactory systems.

The number of OBPs detected in *B. tabaci* (eight) is substantially lower than that detected in *Drosophila melanogaster* (52), *Anopheles gambiae* (69), *Bombyx mori* (44), *Tribolium castaneum* (50) and *Apis mellifera* (21) (Table S4) (Lynch & Conery, 2000; Zhou, 2010; Vieira & Rozas, 2011; Xue *et al*., 2014; Benoit *et al*., 2015; Mesquita *et al*., 2015; Pelosi *et al*., 2018). The relatively low number of OBPs in *B. tabaci* has several possible explanations. First, species in the Hemiptera may in general have fewer OBPs than species in other orders (Table S4). However, Hemiptera species do not encounter a less diverse odorant complexity compared to other insect species. There's parallel relation between the number of OBPs and the degree of diversity of odor space. OBPs play important roles as carriers for odors through the sensillar lymph to transmembrane chemoreceptors. The odorant receptors (ORs) interact with odors, initiate downstream signaling, and ultimately lead to behavioral responses (Leal, 2013). Therefore, the number of ORs and OBPs is related to the diversity of odor. Second, the low number of OBPs may be compensated for the substantial number of CSPs in *B. tabaci*. Both OBPs and CSPs solubilize and transport odor molecules (Pelosi *et al*., 2006, 2014, 2018). Compared to *Cimex lectularius*, *Nilaparvata lugens* and *Acyrthosiphon pisum*, the number of OBPs in *B. tabaci* was lower while the number of CSPs was higher (Table S4). The combined number of OBPs and CSPs is similar in *A. pisum*, *C. lectularius*, *N. lugens* and *B. tabaci*.

We found that *BtabBOBP1*, *BtabBOBP6* and *BtabBOBP8* were arranged on scaffold 135 with the same transcription orientation. Our analysis also revealed that 10 CSPs were organized into three clusters on three scaffolds. This suggests that OBPs and CSPs may have undergone gene duplications in the genome. The duplication of individual genes has long been recognized as a major source of evolutionary novelties, including new genes and gene functions (Hanada *et al*., 2008; Kaessmann, 2010). Several OBPs and CSPs are organized in large clusters and are localized on the same scaffold in *Rhodnius prolixus*, *C. lectularius* and *Papilio xuthus* (Ozaki *et al*., 2008; Benoit *et al*., 2015; Mesquita *et al*., 2015). A previous study has shown that silkworm OBPs have undergone rapid evolution following a complex set of gene duplication events, which was hypothesized to have enhanced the ability to detect diverse sets of odorants (Gong *et al*., 2009). Therefore, we suspect that the OBPs and CSPs in *B. tabaci* evolved to detect specific odorants important to the species.

To explore OBP and CSP functions, we investigated expression patterns in different developmental stages, sexes and tissues. Some BtabBCSPs had unique expression patterns with respect to developmental stage, which suggests stage‐related functions. *BtabBCSP2 and BtabBCSP3*, for example, were mainly expressed in adults, while *BtabBOBP4* and *BtabBOBP5* were highly expressed in nymphs. *B. tabaci* nymphs normally feed on only one individual plant, while adults may disperse and feed on multiple plants. Therefore, *BtabBCSP2* and *BtabBCSP3* may be involved in the perception of plant volatiles. In *N. lugens*, an OBP (*NlugOBP3*) with a similar expression pattern as *BtabBOBP4* and *BtabBOBP5* is hypothesized to have non‐olfactory functions, such as the transporting of juvenile hormone (He *et al*., 2011). Perhaps *BtabBOBP4* and *BtabBOBP5* are involved in the metamorphosis of *B. tabaci* nymphs into adults. *BtabBCSP4* is also highly expressed in eggs, which suggests that it is associated with *B. tabaci* development. In *Locusta migratoria*; 17 OBPs are abundantly expressed in female reproductive organs, and CSP91 was distinctly expressed in male organs (Jean‐François, 2014). In our study, the expression of *BtabBOBP2*, *BtabBOBP3*, *BtabBOBP8*, *BtabBCSP8*, *BtabBCSP10* and *BtabBCSP12* significantly differed between males and females, suggesting that these genes may be related to reproduction and mating. For *BtabBCSP10* and *BtabBCSP12*, based on their high expression levels in different tissues, we can speculate that *BtabBCSP12* has a potential function in recognition of semiochemicals and *BtabBCSP10* has a potential function in reproduction. Moreover, our study showed that expression of *BtabBOBP2*, *BtabBOBP3* and *BtabBOBP8* was biased toward the head. In *S. litura*, female antennae‐biased expression of two OBP genes is consistent with their binding to the sex pheromones and plant volatiles with different binding affinities (Liu *et al*., 2015). Therefore, these three OBPs in *B. tabaci* are important to study further.

Finally, the OBP and CSP sequences and gene expression data presented in this report provide a foundation for the further study of olfactory functions in *B. tabaci*. In addition, the comparison of *B. tabaci* OBPs and CSPs with those of other insect species may provide insight into the evolution of insect chemosensory mechanisms and environmental adaptation.

## Disclosure

The authors declare no conflict of interest.

## Supporting information


**Table S1** Primers used for polymerase chain reaction analysis of chemosensory proteins (CSPs).
**Table S2** Primers used for polymerase chain reaction analysis of odorant‐binding proteins (OBPs).
**Table S3** Currently available odorant‐binding proteins (OBPs) and chemosensory proteins (CSPs) of *Bemisia tabaci*.
**Table S4** Numbers of validated peripheral chemoreception genes in insects.
**Fig. S1** Alignment of *B. tabaci* odorant‐binding proteins (OBPs). Full‐length amino acid sequences of *Bemisia tabaci* OBPs were aligned by ClustalW and edited using BoxShade. Pink boxes show conserved cysteines, and blue boxes are features of Plus‐C. The conserved Cys residues are indicated. Shading indicates sequence identity >70%.
**Fig. S2** Alignment of *B. tabaci* chemosensory proteins (CSPs). Full‐length amino acid sequences of *Bemisia tabaci* CSPs were aligned by ClustalW and edited using BoxShade. Pink boxes show conserved cysteines. The conserved Cys residues are indicated. Shading indicates sequence identity >70%.
**Fig. S3** The computational pipeline used to identify the odorant‐binding proteins (OBPs) and chemosensory proteins (CSPs) in two MEAM1 *Bemisia tabaci* genomes (Chen et al., 2016, and another unpublished MEAM1 genome, FTP: http://111.203.21.119/download/B.gene.v3.cds.fa) and MEAM1 antenna transcriptome (FTP: http://111.203.21.119/download/B/antenna.fasta).
**Fig. S4** Alignment and motif analysis of odorant‐binding proteins (OBPs) between *Bemisia tabaci* MEAM1 and MED. (A) Sequence alignment and motif information of four different full‐length OBPs (OBP1, OBP4, OBP5 and OBP8) between *B. tabaci* MEAM1 and MED. Pink boxes in alignment show different sites. (B) Summarized motifs conserved in insect OBPs but motif 5 missing in *B. tabaci*. The protein names and sequences of the 120 OBPs from different species were listed in a supplementary file.
**Fig. S5** Alignment and motif analysis of chemosensory proteins (CSPs) between *Bemisia tabaci* MEAM1 and MED. (A) Sequence alignment and motif information of eight different full‐length CSPs (CSP4, CSP5, CSP7, CSP10, CSP11, CSP13, CSP16 and CSP17) between *B. tabaci* MEAM1 and MED. Pink boxes in alignment show different sites. (B) Motifs discovered in insect CSPs. The protein names and sequences of the 64 CSPs from different species were listed in a supplementary file.
**Supplementary file** Protein names and sequences of the odorant‐binding proteins (OBPs) and CSPs used in this analysis.
