# Supplementary material for: Genome‐wide analysis of odorant‐binding proteins and chemosensory proteins in the sweet potato whitefly, Bemisia tabaci
Source: Insect Sci. 2018 Mar 26;26(4):620–34. doi: 10.1111/1744-7917.12576 (PMC7380034; doi:10.1111/1744-7917.12576)
Supplement: Supplementary file 1 — Table S1 Primers used for polymerase chain reaction analysis of chemosensory proteins (CSPs). Table S2 Primers used for polymerase chain reaction analysis of odorant‐binding proteins (OBPs). Table S3 Currently available odorant‐binding proteins (OBPs) and chemosensory proteins (CSPs) of Bemisia tabaci. Table S4 Numbers of validated peripheral chemoreception genes in insects. Fig. S1 Alignment of B. tabaci odorant‐binding proteins (OBPs). Full‐length amino acid sequences of Bemisia tabaci OBPs were aligned by ClustalW and edited using BoxShade. Pink boxes show conserved cysteines, and blue boxes are features of Plus‐C. The conserved Cys residues are indicated. Shading indicates sequence identity >70%. Fig. S2 Alignment of B. tabaci chemosensory proteins (CSPs). Full‐length amino acid sequences of Bemisia tabaci CSPs were aligned by ClustalW and edited using BoxShade. Pink boxes show conserved cysteines. The conserved Cys residues are indicated. Shading indicates sequence identity >70%. Fig. S3 The computational pipeline used to identify the odorant‐binding proteins (OBPs) and chemosensory proteins (CSPs) in two MEAM1 Bemisia tabaci genomes (Chen et al., 2016, and another unpublished MEAM1 genome, FTP: http://111.203.21.119/download/B.gene.v3.cds.fa) and MEAM1 antenna transcriptome (FTP: http://111.203.21.119/download/B/antenna.fasta). Fig. S4 Alignment and motif analysis of odorant‐binding proteins (OBPs) between Bemisia tabaci MEAM1 and MED. (A) Sequence alignment and motif information of four different full‐length OBPs (OBP1, OBP4, OBP5 and OBP8) between B. tabaci MEAM1 and MED. Pink boxes in alignment show different sites. (B) Summarized motifs conserved in insect OBPs but motif 5 missing in B. tabaci. The protein names and sequences of the 120 OBPs from different species were listed in a supplementary file. Fig. S5 Alignment and motif analysis of chemosensory proteins (CSPs) between Bemisia tabaci MEAM1 and MED. (A) Sequence alignment and motif information of eig [file INS-26-620-s001.docx]

**Supplementary materials**

**Table S1** Primers used for PCR analysis of CSPs.

| Gene | Primer sequence^a^ (5′ to 3′) |
| --- | --- |
| BtabBCSP1 | F-GTCCCTTCCACAAATAATGCG |
|  | R-AATCACCCTCCAAATCTGCGT |
| BtabBCSP2 | F-TCGAATTGGTAATCGGTCAT |
|  | R-ATTGGAAAGGGTCTCGTTGT |
| BtabBCSP3 | F-GCTGATGTGGCTTGGTTCT |
|  | R-TTCTGGTCATAGGGCGTTT |
| BtabBCSP4 | F-AGTTTTGCCCGATGCCTGAA |
|  | R-CAACAATGATTAGCCCCTAG |
| BtabBCSP5 | F-GAGCGTTTACCTTTGTCGG |
|  | R-AAGTGTCTTCTTGGTTTCG |
| BtabBCSP6 | F-TCCTTTCGTTTCGTCGTCTC |
|  | R-TCACTCCGTGGCATTATTTT |
| BtabBCSP7 | F-AAGATGGCTGCCCACGGTGTT |
|  | R-CGCGATTTTCCGAAAAGTTCC |
| BtabBCSP8 | F-CACTCGTTTCAAGGTCACTCCA |
|  | R-ATTAGGTCTATTTACGGCTCCC |
| BtabBCSP9 | F-ATAGTGATTGGCGTATTCG |
|  | R-TTGGAGTTTTGGCAAACCC |
| BtabBCSP10 | F-ACATGCGCTCTGCTGTGCTA |
|  | R-TTCCAGTGATGAGTCCGTGA |
| BtabBCSP11 | F-TAGAGGGCAGGCGTCAACC |
|  | R-TCGTGCGAATCTCGGAGTG |
| BtabBCSP12 | F-CGTTTGGGCGTCTTGATG |
|  | R-GCAACTCAGACCGGGGAC |
| BtabBCSP13 | F-TGTGGCTACAGCTCAATCG |
|  | R-GTCTGGTGAGCCGCAGATT |
| BtabBCSP14 | F-ATTTCTTGGGAGCGTCTT |
|  | R-CCTCCGTCGTAGAGCATC |
| BtabBCSP15 | F-TTGCCGCTTCGTTGTCTATCC |
|  | R-CCCGTGCCCTCCAGTCTTATT |
| BtabBCSP16 | F-ATGCCCTGTACGAGTGAC |
|  | R-AAGTTTTACCCAGCCACA |
| BtabBCSP17 | F-CGTGGTTCTGCACTTTGG |
|  | R-CTTACCCGGTGTTGTTCG |
| BtabBCSP18 | F-GCAATCACCCTCCAAATC |
|  | R-AAGGCAGTATAATGCAAACG |
| BtabBCSP19 | F-CTAATTTCATGCCATTGTTG |
|  | R-ATCCATTCCAGTGCTTCAGT |

**Table S2** Primers used for PCR analysis of OBPs.

| Gene | Primer sequence^a^ (5′ to 3′) |
| --- | --- |
| OBP1 | F-GTCACTAGCAACTTTGGA |
|  | R-TCTGTCTGGAGAATAGGC |
| OBP2 | F-CTTTGGGCTCTGATACGG |
|  | R-TCATCACAACGTCCTCCT |
| OBP3 | F-TCGAAATGAGTGCGAGTA |
|  | R-GAATGAAACGAAGGTGG |
| OBP4 | F-CCTGAGTGATCTGTGAAGTAA |
|  | R-AGGTAGTGTAGACGAGGGTTG |
| OBP5 | F-CCCCGAGATGTCGTCCCT |
|  | R-GCCCACTTGGCGGTTGAA |
| OBP6 | F-AAAGTCCGTGCGAGAAGA |
|  | R-GCAGTTGCCGATGCGTAA |
| OBP7 | F-GGGGTATGGGCGGAGGGGGTA |
|  | R-TCGGCAGTTTCCTAGTTTGA |
| OBP8 | F-GGTCATCCTCGCAGTTTC |
|  | R-TGTTGCTGATTTCGGTTT |

**Table S3** Currently available OBPs and CSPs of *Bemisia tabaci*.

|  | **MEAM1** | **MEAM1** | **MED** | **MED** |
| --- | --- | --- | --- | --- |
| Searched data | Genome and antennae transcriptome | Homology-based clone | Head transcriptome | Homology-based cloning and sequencing |
| Num. of obtained OBP | 8* | 0 | 8 | 1 |
| Num. of obtained CSP | 19* | 3 | 13 | 3 |
| Ref. | This study | Wang *et al*., 2016a; Liu *et al*., 2016; Li *et al*., 2012; Liu *et al*., 2014. | Wang *et al*., 2017. | Wang *et al*., 2016a; Liu *et al*., 2016; Li *et al*., 2012; Liu *et al*., 2014; Wang *et al*., 2016b. |

An asterisk (*) indicates that the OBP or CSP sequences obtained in this study completely cover the previous sequences (Wang *et al*., 2016a; Liu *et al*., 2016; Li *et al*., 2012; Liu *et al*., 2014; Wang *et al*., 2017; Wang *et al*., 2016b).

**Table S4** Numbers of validated peripheral chemoreception genes in insects.

| **Order** | **Species** | **OBP** | **CSP** | **Total** |
| --- | --- | --- | --- | --- |
| Hymenoptera | *Apis mellifera* | 21 | 6 | 27 |
| Orthoptera | *Locusta migratoria* | 22 | 70 | 92 |
| Lepidoptera | *Bombyx mori* | 44 | 20 | 64 |
| Coleoptera | *Tribolium castaneum* | 50 | 21 | 71 |
| Diptera | *Drosophila melanogaster* | 52 | 4 | 56 |
|  | *Anopheles gambiae* | 69 | 8 | 77 |
| Hemiptera | *Acyrthosiphon pisum* | 16 | 12 | 28 |
|  | *Nilaparvata lugens* | 11 | 17 | 28 |
|  | *Bemisia tabaci* | 8 | 19 | 27 |
|  | *Rhodnius prolixus* | 19 | 27 | 46 |
|  | *Cimex lectularius* | 11 | 16 | 27 |

Numbers of OBP and CSP genes in insects above. (Pelosi & Rozas, 2017; Vieira *et al*., 2011; Zhou, 2010; Lynch & Conery, 2000; Xue *et al*., 2014; Benoit *et al*., 2015; Mesquita *et al*., 2015).

**Figure S1. Alignment of *B. tabaci* MEAM1 OBPs.** Full-length amino acid sequences of *B. tabaci* OBPs were aligned by ClustalW and edited using BoxShade. Pink boxes show conserved cysteines, and blue boxes are features of Plus-C. The conserved Cys residues are indicated. Shading indicates sequence identity>70%.

**Figure S2.** **Alignment of *B. tabaci* MEAM1 CSPs.** Full-length amino acid sequences of *B. tabaci* CSPs were aligned by ClustalW and edited using BoxShade. Pink boxes show conserved cysteines. The conserved Cys residues are indicated. Shading indicates sequence identity > 70%.

**Figure S3.** **The computational pipeline used to identify the OBPs and CSPs in two MEAM1 *B. tabaci* genomes** (Chen et al., 2016, and another unpublished MEAM1 genome, FTP: http://111.203.21.119/download/B.gene.v3.cds.fa) and MEAM1 antenna transcriptome (FTP: http://111.203.21.119/download/B_antenna.fasta).


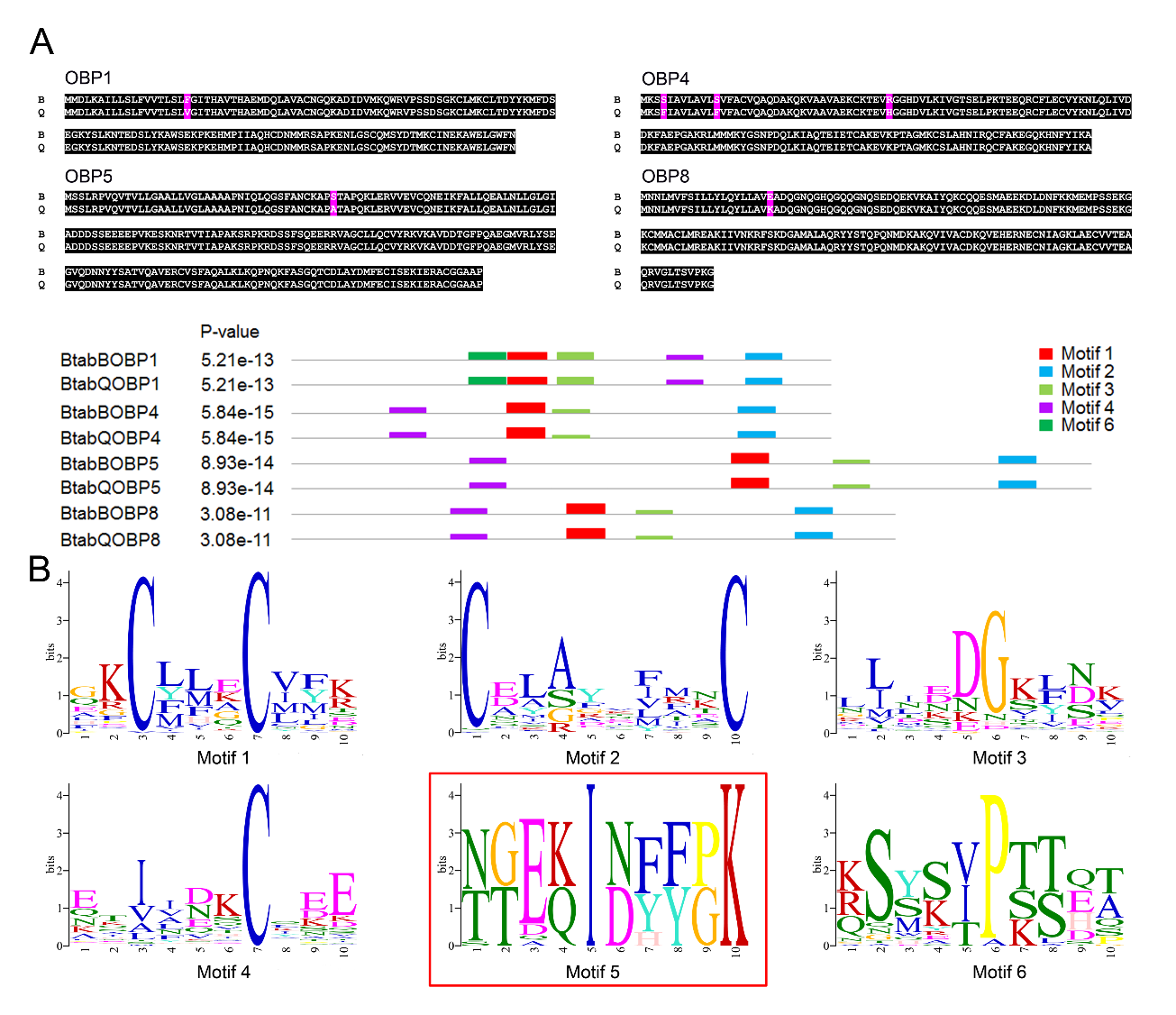


**Figure S4.** **Alignment and motif analysis of OBPs between *B. tabaci* MEAM1 and MED.** (A) Sequence alignment and motif information of four different full-length OBPs (OBP1, OBP4, OBP5 and OBP8) between *B. tabaci* MEAM1 and MED. Pink boxes in aliganment show different sites. (B) Summaried motifs conserved in insect OBPs but motif 5 mssing in *B.tabaci*. The protein names and sequences of the 120 OBPs from different species were listed in Supplementary file.

**
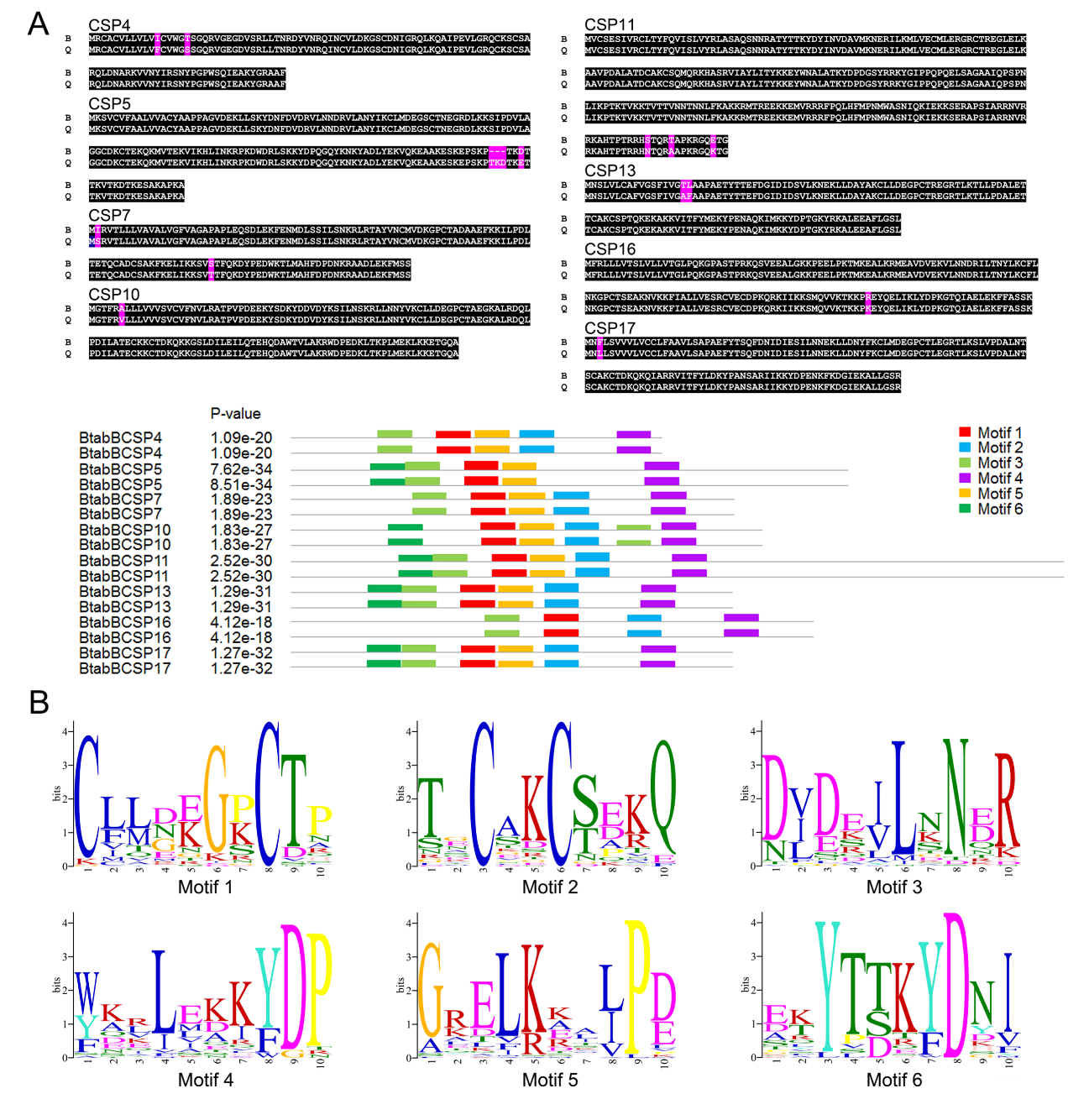
**

**Figure S5.** **Alignment and motif analysis of CSPs between *B. tabaci* MEAM1 and MED.** (A) Sequence alignment and motif information of eight different full-length CSPs (CSP4, CSP5, CSP7, CSP10, CSP11, CSP13, CSP16 and CSP17) between *B. tabaci* MEAM1 and MED. Pink boxes in aliganment show different sites. (B) Motifs discovered in insect CSPs. The protein names and sequences of the 64 CSPs from different species were listed in Supplementary file.

**Supplementary file**

>BtabBOBP1

MMDLKAILLSLFVVTLSLFGITHAVTHAEMDQLAVACNGQKADIDVMKQWRVPSSDSGKCLMKCLTDYYKMFDSEGKYSLKNTEDSLYKAWSEKPKEHMPIIAQHCDNMMRSAPKENLGSCQMSYDTMKCINEKAWELGWFN

>BtabBOBP2

MAMRALIGLFLVAAFFVQRCKAESTTPIPMPEEEDELPARFRSRVVRSASEEKSKEDSSEECGPPQHHHPHPHKPPPECCKGLPRPCNPTEDKKAWDTCVNKLSKKNPTTPQPTTQANGGKPGHGPMHGIDKQCMVECVYNETGLLTPDMKLQEAKISQKLTDKLKDTKFASMAPSVISKCFEKAKNAKDPKECKSGAGEFSKCVNRELFMNCPTESWTNSEECQKMKEKLTKCPNMPVPMSPPPKP

>BtabBOBP3

MSASTARCVGVVAAVFVLTIIAAVAAYDFEDAAFNARLIGDLDEYAVSSARNRRDADLDVDEFFQCKQRNQKVCCGKSSLFKNFGDKDKTAGKACYREIAEKLKRKDDLDEDLMDIFSCEQIKQMKKKHFCIHECIGKRKNLLNADGSINVLNMRTYLKKELFTEEWQQKVGDKAMEKCFNEKYVSSWPDSDSSDVKCNPISVQFQHCLWKQIELNCPDSEWKDTRRCNKIKEYLRKQELSQKNKISKN

>BtabBOBP4

MKSSIAVLAVLSVFACVQAQDAKQKVAAVAEKCKTEVRGGHDVLKIVGTSELPKTEEQRCFLECVYKNLQLIVDDKFAEPGAKRLMMMKYGSNPDQLKIAQTEIETCAKEVKPTAGMKCSLAHNIRQCFAKEGQKHNFYIKA

>BtabBOBP5

MSSLRPVQVTVLLGAALLVGLAAAAPNIQLQGSFANCKAPSTAPQKLERVVEVCQNEIKFALLQEALNLLGLGIADDDSSEEEEPVKESKNRTVTIAPAKSRPKRDSSFSQEERRVAGCLLQCVYRKVKAVDDTGFPQAEGMVRLYSEGVQDNNYYSATVQAVERCVSFAQALKLKQPNQKFASGQTCDLAYDMFECISEKIERACGGAAP

>BtabBOBP6

MQGEVCFALLVVCLLHVTFIPDVETAVSEQMIKQMKDMHKKTCMARSGVKKEGLAKFMEEGVNDDPAFKKYTLCMLKNMQGFKNGKIAIKDIENQAKAMLPPPLRDAILDATSKCSNTGGDTPEDITYNFSKCSHKANVKSVMII

>BtabBOBP7

MGGGGRPWSQNTQDSGRGGGNGGGRHQSNQYGNQNTQDSGRGGGNGGGRHQSNQYGNQNNDRYCSSGYQNSDRDRPRFSSSGSGGGGGGGNDDDFYTKTQSEQRFRGDGGGGGRTRPGQYRVKVQYGSGQGGGPDSRAYPLGRKRRATKIISNNAKNATGRNPTSSETGKTSLLEELDACVVQCIFRQMEMLTEDARPDKNSVISVMTQRIRDPELKEFIQESIEECFDIIESDAEGGKCEYSKNFAMCLEEKGRRNCEDWEEAAQINRFKGIQSGMSPPDTTNNYNPPNFNNFNSNNQGNRQNAFYNYNGK

>BtabBOBP8

MNNLMVFSILLYLQYLLAVEADQGNQGHQGQQGNQSEDQEKVKAIYQKCQQESMAEEKDLDNFKKMEMPSSEKGKCMMACLMREAKIIVNKRFSKDGAMALAQRYYSTQPQNMDKAKQVIVACDKQVEHERNECNIAGKLAECVVTEAQRVGLTSVPKG

>AglyOBP2

MKVSAATAVLVALVATVQSSDPCNISTCYKSGTTKPPTTVTPTRLPVQSSSTPTSHQQTTYAKDHVHSSTATKSGVNTTATTTSGASVNGTERTTVVKSSSGVAGNVTTPKPTMTDGHLALKQKLNTIAVKCKDELHAPQEIMALVSNTVVPQNEQQRCYLECVYKNLNLIKNNKFSVDDGKAMAKIRFANQPEEHKKAVTIIETCEKEAIIDPKTTEKCAAGRVIRNCFVKNGEKINFFPKA

>AglyOBP3

MISSTFYTSLMFGIVMLISCSFGRFTTEQIDHYGKACNATEDDLVVVKSYKVPTSDTGKCLMKCMISKLGLLNDDGSYNKTGMEAGLKKYWSEWSTDTIESINNKCYEEALLVSKDIIATCNYAYVVMACLNKQLDLDKST

>AglyOBP4

MRGNYSLVVFLLFGFGLLEIYCQKQETSGKCRAPDKAPLNLEIIINICQEEIKSALLQEALDILNDGTLEQNTPSYSRSKRDADEDLSNEERRVAGCLLQCVYKKVKAVDETGFPVVDGLMKLYNEGVQDRNYYMATLSAVRHCISIAQQLKQQQPSKSFDDGQTCDLAYEMFECVSEKIEENCGVENKSNN

>AglyOBP5

MKRFGDKDKVAADECYAQVAEKFATVTATTPKQDLFSGEAVKITKKKQFCLHECIGKKNKLLTEDGSLNKTFIADYAMKSVFKEQWQKQIGQKALDKCLEETYIPWPAEETENKCNPVYVQFQHCLWLEYESNCPDNKIKLTKKCEKTRNRYRMQKSTSN

>AglyOBP6

MQKVVFLCIFAIICQTVFTVGFERTWILRQKRVTNDDECRTLIPSSEKKLPTCCQMPNILPGLDNAWEVCFEKFKQFKDKHATKEYKEMAHGNEPPCLFQCVFMQSGLTTSDGKVNEDAVIKKMAEGMDNDEKWKSIWRNTFNKCLNDVKQEDKEQIKMTNTPTGRLMKCFLRDLYMNRPKNVWVESSECSNLKDLVEKCPKMPPPVFKSPPKLI

>AglyOBP7

MVARKRMYMLPATVLLAVVAATILKDSDAYLSEEAIKKTQKMLKNVCSKKHSVEEEVFTDIKKGIFPENNNNIKCYFACNFRTMQMVNQKGILDKKMFKDKMTMLAPPNVLAILLPPIEQCIGNDKDTEICRSSYNFIKCAHRVDPKSLEFLPL

>AglyOBP8

MFAFKVACLCLSVAVVFGENNQQNSNDRSASIFQSCISETKLSGDALKGFRSMSIPKTQAEKCMMGCLMRKVNVINNGKFSVEEATKVAQKYYGTNETMMKKAKDLIDVCAKKAQSTTEECALAGIVTTCIVEEAQKAGLTGGPGSRSKRTVSPKFRHSIV

>AglyOBP9

MIIKKTLLVSGFVLFGCMFSINKAADDADAKDKELMSKLITVAFKCFKDADWGTCGEMITTKYDITQAKYKQCTCHMACAGEDLGLINSNGQPEPAKFLEYVKRINNSVIKSQLQHIYDKCQNVKGTEKCDLAEQFAICAFKESPEMKERVTKLIEMLVKMKPKSK

>AglyOBP10

MEHLRGTNVVFAIVMALLVVQSSTRPQPDELDDIKKTLYNACSEKFPLTEEIKNNVKNSIVIDDQNFKCFLRCCFDEMSLIDEDGIIDGESLAAMAVDKIKPVAEKIVHDCLPAGKQEKQDGCEASFKFFSCGIKLNPLTIELLPLQ

>AglyOBP11

MISSTFYTSLLFGIAMLISCSFGRFTTEQIDHYGKACNATEDDLVIVKSYKVPTSDTGKCLMKCMISKLGLLNDDGSYNKTGMEAGLKKYWSEWSTDTIESINNKCYEEGGTICFILNRFVT

>AfabOBP2P

SDPCNISTCYQSGTTKPPTTVTPTRLPVQSSSTPTSHQQTTYAKDHVHSSTATKSGVNTTATTTSGASVNGTERTTVVKSSSGVAGNLTTPKPTMTEGHVALKQKLNTIAVKCKDELHAPQEIMALVSNTVVPQNEQQRCYLECVYKNLNLNKNNKFSVDDGKAMAKIRFANQPEEHKKAVTIIETCEKEAIIDPKTTEKCAAGRVIRNCFVKNGEKINFFPKA

>AfabOBP8P

ENNQQNSNDRSATIFQSCISETKLSGDALKGFRSMSIPKTQAEKCMMGCLMRKVNVINKGKFSVEEATKVAQKYYGTNETMMKKAKDLIDVCAKKAQSTTEECALAGIVTTCIVEEGQKVGLTGGPGGRSRRTVSPKFRRNSM

>AcraOBP2P

SDPCNISTCYKSGTTKPPTTVTPTRLPVQSSSTPTSHQQTTYAKDHVHSSTATKSGVNTTATTTSGASVNGTERTTVVKSSSGVAGNATTPKPTMTEGHVALKQKLNTIAVKCKDELHAPQEIMALVSNTVVPQNEQQRCYLECVYKNLNLIKNNKFSVDDGKAMAKIRFANQPEEHKKAVTIIETCEKEAIIDPKTTEKCAAGRVIRNCFVKNGEKINFFPKA

>BbraOBP3

MISSTFYITLLFGIAMIISCSYGRFTTDQIDYYGKACNASEDDLVVVKSYKVPSTETGKCLMKCMITKLGLLNDDGSYNKTGMEIGLKKYWSEWSTEKIEAINNKCYEEALLVSKEVIATCNYSYTVMACLNKQLDLDKST

>TsalOBP1P

ESDQVPMNSSAAVENCLLETNMTRDEFEDMLTSPNARELTILKSHAHKCMLGCVMRKNHIVNDGVVSKEVLSKYVLNFYGRPDYKRRLIIKDVEHIVDVCAKKVADESETDECELAATLVTCIVLEANKAGLVDDPARQI

>DplaOBP3

MISSTFYITSVFGIAMLISCGYGRFTTDQIDYYGKACNASEDDLVVVKSYKVPSTETGKCLMKCMITKLGLLNDDGSYNKTGMEAGLKKHWSEWSTEKIENINNKCYEEALLVSKEVVATCNYSYTVMACLNKQLDLDKST

>PsalOBP1P

ESDQVPMNSSAAVENCLLETNMTRDEFEDMLTSPNARELTILKSHAHKCMFGCVMRKNHIVNDGVVSKEVLSKYVLNFYGRPDYKRRLIIKDVEHIVDVCAKKVADESETDECELAATLVTCIVLEANKAGLVDDPARQI

>PsalOBP2P

SDPCNISTCYKSGTTKPPTTVTPTRLPVQSSSTPTSHPQTTYAKDHAHGSTTVKSGANVTATKDNKATVNGTTERPAVKSTTGAAGNATTLNSTMTEVHVALKQKLNTIAVKCKDELHAPQEIMALASNTVVPQNEQQRCYLECVYKNLNLIKNDKFSVDDGKTMAKIRFAKQPEEYKKAVTIIETCEKEAVIDPKTTEKCAAGRVIRNCFVKNGEKINFFPKA

>PsalOBP4P

QKQETSGKCRAPDKAPLNLEIIINTCQEEIKSALLQEALDILNDGNTEQNTQNQSNRSKRETEEDLTNEERRVAGCLLQCVYKKVKAVDETGFPVVDGLMKLYNEGVQDRNYYIATLSAVRHCISIAQQLKQQQPSKTFDDGQTCDLAYEMFECVSEKIEENCGVENKSNN

>PsalOBP9P

DDADAKDKELMAKLFGVALKCFKDADWGACGEMITTKYDITEPKYKQCTCQMACVGEDLGMINTKGEPEPAKFLEYVKRINNQSIKSQLQHIYDKCQNVKGADKCDLSEQFAICAFKESPALKERVSTLMEMLVKMKPKSK

>PsalOBP10P

STRPQPDELEEVKKSLYNACSSKFPLTEEIRNQAKNGILTEDPNLKCFLRCCFDEMSLIDEDGIIDGETLVAMSMDRIKLITEQAVHNCLKTTKQDGCEASFQFLSCGIKLNPLI

>LeryOBP3P

RFTTEQIDYYGKACNASEDDLAVVKSYKVPSTETGKCLMKCMITKLGLLNDDGSYNKTGMEIGLKKYWSEWSTEKIEAINNKCYEEALLVSKEVVATCNYSYTVMACLNKQLDLDKST

>LeryOBP7P

MVARKRMYNMLPTNVLLTIIAATVLNDCDAYLSEAAIKKTQQMLKSVCSKKYTVEEDVFTNIKKGIFPEDNNNIKCYFSCVFKTMQMINQKGSLDKKIFKEKMSMMAPPSVYNILLPAIEQCIGKDNGEELCQASYNFIKCAHHIDPKSLEFLPL

>MvicOBP1P

EIDQVPINSSAAVENCLLETNMTRDEFEDMLTSPNARELTILKSHAHKCMFGCVMRKNHIVNDGVVSKEVLSKYVLNFYGRPDYKRRLIIKDVEHIVDVCAKKVADESETDECELAATLVTCIVLEANKAGLVDDPARQI

>MvicOBP2P

SDPCNISTCYKSGTTKPPMSVTPTRLPVQSSSTPTSHPQTTYAKDHSHGSTTTKSGANATATTASGASVNGTERPAVVKSSAGVTGNLTTPKPTMTEGHVALKQKLNTIAVKCKDELHAPQEIMALVSNTVVPQNEQQRCYLECVYKNLNLIKNNKFSVEDGKAMAKIRFANQPDEHKKAVTIIETCEKEAVIDPKTTEKCAAGRVIRNCFVKNGEKINFFPKA

>MvicOBP5P

DAGHHRRGKELLDTEDSDFFRCKQASRKSCCGPENAMKRFGDKDKVAADECYAQVAEKFATVTATTPKQDLFSADAVKITKKKQFCLHECIGKKNHLLTEDGSLNKTFIADYAMKSVFKEQWQKPVGLKALEKCLEETYIPWPAEDKENVCNPVYVQFQHCLWLQYESNCPANKIKITKKCEKTRNRYRMQKSTSN

>MvicOBP8P

ENNQQNSNDRSATIFQSCISETKLSGDALKGFRSMSIPKTQAEKCMMGCLMRKVNVINKGKFSVEEATKVAQKYYGTNETMMKKAKDLIDVCAKKAQSTTEECALAGIVTTCIVEEAQKAGLSGGPGSRSRRTVSPKFRRNSM

>MvicOBP10P

STRPQPDELEEIKKTLYNACAGKFPITEEVKNNAKNSIFLDDQNFKCFLKCCLDEMSLIDDDGIIDGDSLKAMASDKIKPMXEQVVPNCLKNVQQDGLEAAFVFLRXGRG

>MdirOBP1P

EIDQVPINSSAAVENCLLETNMTRDEFEDMLTSPNARELTILKSHAHKCMFGCVMRKNHIVNDGVVSKEVLSKYVLNFYGRPDYKRRLIIKDVEHIVDVCAKKVADESETDECELAATLVTCIVLEANKAGLVDDPARQI

>MdirOBP2P

SDPCNISTCYKSGTTKPPMAVTPTRLPVQSSSTPTSHPQTTYAKDHVHGSTTIKSGANATATTASGASVNGTERPTVVKSSAGVIGNSTTPKPTMTEGHVALKQKLNTIAVKCKDELHAPQEIMALVSNTVVPQNEQQRCYLECVYKNLNLIKNNKFSVEDGKAMARIRFANQPEEHKKAVTIIETCEKEAIIDPKTTEKCAAGRVIRNCFVKNGEKINFFPKA

>MdirOBP3P

RFTTEQIDYYGKACNASEDDLVVVKSYKVPSTETGKCLMKCMITKLGLLNDDGSYNKTGMEAGLKKYWSEWSTEKIENINNKCYEEALLVSKEVVATCNYSYTVMACLNKQLDLDKST

>MdirOBP4P

QKQETSGKCRAPDKAPLNLEIIINICQEEIKSALLQEALDILNDGNLEQNTPASYSSRSKREADEDLTNEERRVAGCLLQCVYKKVKAVDETGFPVVDGLMKLYNEGVQDRNYYMATLSAVRHCISIAQQLKQQQPSKSFDDGQTCDLAYEMFECVSEKIEENCGVENKSNN

>MdirOBP5P

DAGHHRRGKELLDTEDSDFFRCKQASRKSCCGPENAMKRFGDKDKVAADECYAQVAEKFATVTATTPKQDLFSAEAVKITKKKQFCLHECIGKKNNLLTEDGSLNKTFIADYAMKSVFKEQWQKQVGQKALDKCLEETYIPWPAKNKENVCNPVYVQFQHCLWLQYESNCPANKIKITKKCEKTRNRYRMQKSTSN

>MdirOBP6P

PNILPNSNSTWAKCFETFKQFKDKPETKEYKEMAHGKEPPCLFQCIFVQSGLTTSDGKLNEDAITKKMSEGINNDEKWKSTWQNSLNKCFDDVKQEDKKQILIMNTPAGRLMKCFLRDMYMSCPKSVWVESSECLNMKDLVQKCPEMPPPVFKSPPKLI

>MdirOBP8P

ENNQQNSNDRSATIFQSCISETKLSGDALKGFRSMSIPKTQAEKCMMGCLMRKVNVINKGKFSVEEATKVAQKYYGTNGTMMKKAKDLIDVCAKKAQSTTEECALAGIVTTCIVEEAQKAGLSGGPGSRSRRTVSPKFRRNSM

>MperOBP3P

RFSTEQIDYYGKACNASEDDLVVVKSYKVPTTETGKCLMKCMITKLGLLNDDGSYNKTGMEAGLKKYWSEWSTEKIEAINNKCYEEALLVSKEVIATCNYSYTVMACLNKQLDLDKST

>MperOBP4P

QKQEPSGKCRAPDKAPLNLEIIINICQEEIKSALLQEALDILNDGNLEQNTPSYSSRSKREADEDLTNEERRVAGCLLQCVYKKVKAVDETGFPVVDGLMKLYNEGVQDRNYYMATLSAVRHCISIAQQLKQLQPSKSFDDGQTCDLAYEMFECVSEKIEENCGVENKSNN

>MperOBP6P

PNILPGLDSTWEKCYEKFIQFKDKPETKEYKEMSHGKEPPCLFQCIFMESGLTTNDGKLNEDAITKKMTEGINNDEKWKSTWKKSLDKCFDDVKQEDKKQILIMNTPAGRLMKCFLRDIYMNCPENVWVESSECLNVKNLVQKCPEMPPPVFQSAPKLI

>MperOBP7P

YLSEAAIKKTQQMLKTVCSKKHSVEEDVFTDIKKGIFPENNNNIKCYFACNFKTMQMINQKGTLDKKLFKDKMSMMAPPNIYNILLPAIEQCIGIDKGEELCQSSYNFIKCAHRVDPKSLEYLPL

>MperOBP8P

ENNQQNSSDRSATIFQSCIAETKLSGDALKGFRSMSIPKTQAEKCMMGCLMRKVNVINKGKFSVEEATKVAQKYYGTNETMMKKAKDLIDVCAKKAQSTTEECALAGIVTTCIVEEAQKAGLAGGPGSRSRRTVSPKFRRNSM

>NribOBP2P

SDPCNISTCYKSGTTKPPMTVTPTHLPVQSSSTPTSHPQTTYAKDHVHGSTTTKSGANATATTASGASVNGTERPAVVKSSAGVTGNFTTPKPTMTEGHVALKQKLNTIAVKCKDELHAPQEIMALVSNTVVPQNEQQRCYLECVYKNLNLIKNNKFSVEDGKAMARIRFANQPEEHKKAVTIIETCEKEAVIDPKTTEKCAAGRVIRNCFVKNGEKINFFPKA

>NribOBP3P

RFTTEQIDYYGKACNASEDDLVVVKSYKVPSTETGKCLMKCMITKLGLLNDDGSYNKTGMEAGLKKYWSEWSTEKIETINNKCYEEALLVSKEVVATCSKSHDRKACLNQDPDLDKST

>NribOBP5P

DAGHHRRGKELLDTEDSDFFRCKQASRKSCCARKNAMKRFGDKNKVAADECYAQVAEKFATVPATTHKQDLFSAEAVKITKKKQFCLHECIGKKNNLLTEDGSLNKTFIADYAMKSVFKEQWQKEVGQKALDKCLEETYIPWPAEDKENVCNPRYVQIQHCLWLLSRRNIPAHKSKITKKCEKTRNRYRMQKSTSN

>NribOBP7P

YLSEAAIKKTQHMLKTVCSKKHSVDEDVFTEIKKGIFPEDNNDIKCYFACNFKTMQLVNQKGYIDKKLFKDKMSIMAPPNVYNILLPVIEQCAGIDKSEELCQSSYNLIKCAHRVNPKSLEFLPL

>NribOBP8P

ENNQQNSNDRSATIFQSCISETKLSGDALKGFRSMSIPKTQAEKCMMGCLMRKVNVINKGKFSVEEATKVAQKYYGTNETMMKKAKDLIDVCAKKAQSTTEECALAGIVTTCIVEEGQKAGLTGGPGGRSRRTVSPKFRRNSM

>RpadOBP2P

SDPCNISTCYKSGTTKPPTTVTPTRLPVQSSSTPTSHQQTTYAKDHAHSSIAAKSGANVTATTASGATVNGTERPTVVKSSPGVAGNATTPKPTMTVEHVALKQKLNTIAVKCKDELHAPQEIMALVSNTVVPQNEQQRCYLECVYKNLNLIXNNKFSVDDGKAMARIRFANQPEEHEKAVTIIETCEKEAIIDPKTTEKCAAGRVIRNCFVKNGEKINFFPKA

>RpadOBP5P

DAGHHRRGKELLDTEDSDFFRCKQASRKSCCGPDNAMKRFGDKDKVAADECYAQVAEKFATTKATTPKQDLFSSEAVKVTKKKQFCLHECIGKKNKLLTEDGSLNKTFIADYAMKSIFKEQWQKQIGQKALDKCLEETYIPWPAEETENKCNPVYVQFQHCMWFEYESNCPSNKIKLTKKCEKTRNRYRMQKSTSN

>RpadOBP10P

STRPQPDEMEEIKKTLYNACSAKFPLTDEIRNNAKNSIVADDQNLKCFLRCCFDEMSMIDEDGIIDGESLVSMTSDKLKIVAKKAVDSCLTADKQDGCEAAFKFISCGIKLNPLIGSATL

>AgosOBP2

MKVSAATAVLVALVATVQSSDPCNISTCYKSGTTKPPTTVTPTRLPVQSSSTPTSHQQTTYAKDHVHSSTATKSGVNTTATTTSGASVNGTERTTVVKSSSGVAGNVTTPKPTMTDGHVALKQKLNTIAVKCKDELHAPQEIMALVSNTVVPQNEQQRCYLECVYKNLNLIKNNKFSVDDGKAMAKIRFANQPEEHKKAVTIIETCEKEAIIDPKTTEKCAAGRVIRNCFVKNGEKINFFPKA

>AgosOBP3

MISSTFYTSLMFGIAMLISCSFGRFTTEQIDHYGKACNATEDDLVIVKSYKVPTSDTGKCLMKCMISKLGLLNDDGSYNKTGMEAGLKKYWSEWSTDTIESINNKCYEEALLVSKDIIATCNYAYVVMACLNKQLKLDNST

>AgosOBP4

MRGNYSLVVFLLFGFGLLEIYCQKQELSGKCRAPDKAPLNLEIIINICQEEIKSALLQEALDILNDGTLEQNTPSYSRSKRDADEDLSNEERRVAGCLLQCVYKKVKAVDETGFPVVDGLMKLYNEGVQDRNYYMATLSAVRHCISIAQQLKQQQPSKSFDDGQTCDLAYEMFECVSEKIEENCGVENKLNNLSQRQV

>AgosOBP5

MKMSANGATMKCVAVAVVLFQMSVIFAEAGHQRRGKELLDTEDSDFFRCKQASRKSCCGPENAMKRFGDKDKVAADECYAQVAEKFATVTATTPKQDLFSGEAVKITKKKQFCLHECIGKKNKLLTEDGSLNKTFIADYAMKSVFKEQWQKQIGQKALDKCLEETYIPWPAEETENKCNPVYVQFQHCLWLEYESNCPDNKIKLTKKCEKTRNRYRMQKSPSNQ

>AgosOBP6

MQKVVFLCIFAIICQTVFTVGFERTWILRQKRMTNDNECRALFPSPEKKLPTCCQMPNILPGLDNAWEVCFEKFKQFKDKHATKEYKEMVHENEPPCLFQCVFMQSGLTTSDGKVNEDAVIKKMAEGMDNDEKWKSIWRNTFNKCLNDVKQEDKEQIKVMNTPTGRLMKCFLRDLYMNCPKNVWVENSECSNLKDLVEKCPKLPPPVFQSPPKLI

>AgosOBP7

MNMLPATVLLAVVAATILKDSDAYLSEEAIKKTQKMLKNVCSKKHSVEEEVFTDIKKGIFPENNNNIKCYFACNFKTMQMVNQKGILDKKMFKDKMTMLAPPNVLAILLPPIEQCIGNDKDTEICQSSYNFIKCAHRVDPKSLEFLPL

>AgosOBP8

MFAFKVACLCLSVAVVFGENNQQNSNDRSASIFQSCISETKLSGDALKGFRSMSIPKTQAEKCMMGCLMRKVNVINKGKFSVEEATKVAQKYYGTNESMMKKAKDLIDVCAKKAQSTTEECALAGIVTTCIVEEAQKAGLTGGPGSRSKRTVSPKFRHSIV

>AgosOBP9

MIIKKTLLVSGFVLFGCMFSINKAADDADTADKELMSKLITVAFKCFKDADWGTCGEMITTKYDITQAKYKQCTCHMACAGEDLGLINSNGQPEPAKFLEYVKRINNSVIKSQLQHIYDKCQNVKGTEKCDLAEQFAICAFKESPEMKERVTKLIEMLVKMKPKSK

>AgosOBP10

MEHLRGTNVMFAIVMALLVVQSSTRPQPDEPDDIKKTLYNACSEKFPLTEEIKNNVKNSMVIDDQNFKCFLRCCFDEMSLIDEDGIIDGESLAAMAVDKIKPVAEKIVHDCLPAGKQEKQDGCEAAFKFFSCGMKLNPLTIELLPLQ

>ApisOBP1P

ESDQVPINSSAAVESCLLETNMTRDEFEDMLTSPNARELTILKSHAHKCMFGCVMRKNHIVNDGVVSKEVLSKYVLNFYGRPDYKRRLIIKDVEHIVDVCAKKVADESETDECELAATLVTCIVLEANKAGLVDDPARQI

>ApisOBP2P

SDPCNISTCYKSGTTKPPMAVTPTHLPVQSSSTQTSHPQTTYAKDHVHGSTTTKSGVNATVTTASGASVNGTEPPAVVKSSAGVTGNSTTPKPTMTEGHVALKQKLNTIAVKCKDELHAPQEIMALVSNTVVPQNEQQRCYLECVYKNLNLIKNNKFSVEDGKAMARIRFANQPEEHKKAVTIIETCEKEAVIDPKTTEKCAAGRVIRNCFVKNGEKINFFPKA

>ApisOBP3P

RFTTEQIDYYGKACNASEDDLVVVKSYKVPTTETGKCLMKCMITKLGLLNDDGSYNKTGMEAGLKKYWSEWSTEKIESINNKCYEEALLVSKEVVATCNYSYTVMACLNKQLDLDKST

>ApisOBP4P

QKQETSGKCRAPDKAPLNLEIIINTCQEEIKSALLQEALDILNDGNVEQNTPNYSSRSKREAEEDLTNEERRVAGCLLQCVYKKVKAVDETGFPVVDGLMKLYNEGVQDRNYYIATLSAVRHCISIAQQLKQQQPSKSFDDGQTCDLAYEMFECVSEKIEENCGVENKSNN

>ApisOBP5P

DAGHHRRGKELLDTEDSDFFRCKQASRKSCCGPENAMKRFGDKDKVAADECYAQVAEKFATVTATTPKQDLFSAEAVKITKKKQFCLHECIGKKNNLLTEDGSLNKTFIADYAMKSVFKEQWQKQVGQKALDKCLEETYIPWPAEDKENVCNPVYVQFQHCLWLQYESNCPANKIKITKKCEKTRNRYRMQKSTSN

>ApisOBP6P

PNILPNLDSTWEKCFETFKQFKDKPETKEYKEMAHGKEPPCLFQCIFMQSGLTTSDGKLNEDAITKKMSEGINNDEKWKSIWQNSLNKCFDDVKQEDKKQILIMNTPAGRLMKCFLRDMYMSCPKNVWVESSECLNMKDLVQKCPEMPPPVFKSPPKLI

>ApisOBP7P

YLSEAAIKKTQQMLKTVCSKKHSVEEDVFTNIKKGIFPEDNNNIKCYFACNFKTMQLINQKGVIDKKMFKDKMSMMAPPNVYKILLPVIEQCTGKDKGEELCQSSYNVIKCAHSVDPKSLEFLPL

>ApisOBP8P

ENNQQNGPSDRSATIFQSCIAETKLSGDALKGFRSMSIPKTQAEKCMMGCLMRKVNVINKGKFSVEEATKVAQKYYGTNEAMMKKAKDLIDVCAKKAQSTTEECALAGIVTTCIVEEAQKAGLSGGPGSRSRRTVSPKFRRDAM

>ApisOBP9P

DDADAKDKELMSKLFTVVFKCFKDADWGTCGEMITTKYDITQAKYKQCTCHMACAGEELGMINASGQPEPAKFLEYVNKINNPDIKSQLQLIYDKCQNVKGSEKCDLAEQFAICAFKESPALKERVSTLMEMLVKMKPKSK

>ApisOBP11

MSSSTFYITLLFGIAMLISCGYGIFTTEQIDYYGKACNASEDDLIVLKSYKVPSTETGKCLMKCMITKLGLLNDDGSYNKTGMEAGLKKYWSEWATEKIETINEKCYEEGNTATLLYHVAIYFTCVSGDYSDVQLLIHCDGMFEQEVGSRQVNLKLLIMLKIGLSEPKR

>ApisOBP12P

DDLVVVKSYKVPTTETGKCLMKCMITKLGLLNDDGSYNKTGMEAGLKKYWSEWSTEKIESINNKCYEEGDTSTLLYHVVIYFTCVKGGSSDVQLLVHCDGMFEQAVGSRQVNL

>ApisOBP13P

CTIHCVFNQLEMLNSNSRPDKYSIVNIMTNQIKDVELKEFIQDSIDECFDTLELDSHNNKCEFSKNFAVCMENKAQRNCDDWDENLSANKINSAGLQDGTNQQDKRKGY

>AlucOBP1

MCSKYFVMLIGLTVYTSAEVINEECKDRNQSSTEYETFYNCCDLESSFNETKSKEKEEAREFCENEFEKANNVSEDEAEPSPSSVRQDCYVDCILKKLGAMSEDYKMDKEKVTKWFMEGTHKDFEEVGKQAMEKCYDKTYSKKHCASRVMGLLWCYSEELVMNCPAKYWDQSEKCTAAKAYMKKCSTNPWRSED

>AlucOBP2

MRSTGSECFEEIDAKLGNKTSLESDMDPYNCEKVKRMKKRHYCMHECKAKKLGVATEEGNLEFPKVKELLLSRVNETWQKDILGQAADTCATSKFDQTWKDDTEEYKCNPQALQFKHCVWKQVEMKCPEEHQNTGRHCKKLRSKISSETSKDIAKETSV

>AlucOBP3

MFSSATLVCLFAVALTQGQLDEDPECRPPHPPGKDDKCCTIPELIVGENMQAMMKQCFEESGMERRPPGPPGSGTPPTPEEIEAHRSAHECVDECFFKAAKFMNSDGEFDLEAMKTAAASVFTGDWAPLGSETIDKCFASAKSQVSASAKCTSGAHRAKKCILRNFIINCPPSAWNDSTDCAALKARLTKCSNAMPPFPHHKH

>AlucOBP4

MEVAACLVLLAALAALTAAVEEGRPLCKAPTTAPRKLEKVINQCQEEIKYALLQEAPSVLGETVGLKTALTRNRSKRETFTGEERRIAGCLLQCVYRKMKALDETGFPTATGLVKIYSEGVEDRNYYLATIQGVQRCLSRELQSRNTNPSIVKAEGYSCDVAYDMFNCVSEQIEQLCGTSP

>AlucOBP6

MYDRFKLFALLALVVSCKSAPPEEPAECKLPESDSAELVKCCKLNVVLDEMADSVGECMKLVKGKPEKGPPVPEGFDCMDTCVFSKLGFAANNKLDAEKLTKKFSELFKGDWSALSDSTLKKCLPMAEGAKGSCASGADVFKFCIVRELYMNCPASSWTKSDLCKANVERLEKCPHSMPFLPGTGIKKN

>AlucOBP7

MNPLILILLVVFAAATRGEEQANALVAKAFNKCFGEFPLGDDEMKEVKDKSTVPSSHNAKCLMACMLKEGRILRGGKYELENAILMADVLNKNDHAATDKAKQLIETCAAQVGTDASADECEFAYKMALCASDEAKKLGVRPPDF

>AlucOBP8

MVLKMKQILVVFVALQVLISTTEAVMTQAQMKQAMKTVRNMCIPKSGVDKEALAKMVEGEFDESDQKLKCYLGCVLGMMQAVKNNKINLTMVKNQISKMLAPEQGQRILAAFEGCATVTGDDNCDLAFKFAKCIYDTDKELLFQAFIVP

>AlucOBP9

MKSFVGLIFAVALVEFASAITKEYHDRAVAAKDACLKKHPSIKESDVQEFLKKHKLPETDDGKCMIACYMEEMNLMADGKINVEEAKKTNSDKYDGEPDNKELADKLIDHCSSQVSPDGMSKCEYAYQISKCGLEYGMKNGLTPPKMYEEQRR

>AlucOBP10

MTYHVFFRKFDLPRISRRVRQCYYHSVPRSLSGSSRRMLEETSQHHPKRRSRVSEKHKLPETDDGECMIACYMEEKNLMADGKINVKEANQTNSDKYDGEPDNKQLAEKLIDHCSSQVSPDGMSKCEYAYQFSKCGLEYGMKNGLTPPKMYEEQRR

>AlucOBP11

MGSQYERTLVGVRYLPIMKRVKFILVLSLLSRCSSAPTDDMAACMQITNEDSASMATCCDYVIPFSNKTMTTCDKKETSGEMSKEFECVQDCLFSSDNVLGADKKFDPVAWRKHATNTISGDWKGVIANSGSNCEGFKKVLAQSMEKKCPTSESDVSFNCMTLQWYMNCPKSAWTSSESCEASKKKLMSCFGPIFENTS

>AlucOBP12

MTCSHFIALLSVVALSLSSGEINEECKDIENLKTQLENFYGCCDFESMIERVVRTEEEVETDRFCREERKKINSTDGKVPLASEGHDCFMECVLKRMGAMGQDFKFIREKLDDFFLRGYPEEVKQAGKLAFDKCLSKNFSKKYCASGINGLMMCLPEELVMNCPANIWSSHESCPIAKEAIKKCPSYRVMIEQE

>AlinOBP1

MNSLIPVLLVVCAAATRADEQTNAMVAKAFNKCREEFPISDDEIGGVREKTTIPESHNAKCLMACMLREGKMLRDGKYEKENALIMADVLNKDDPASADKAKQLVETCAGKVGTDAGGDECEFAYKMAVCAAEEAKKLGVRPPDF

>AlinOBP2

MSLKIQFFVFAAICAACVCAYQEQLKQTIRDCQDGKEVTDDELEEFTKPLIPRNREEKCIMACVMRTYNIISNGHYDPKIAFGILKGILKDHPEKLNKIKEVMDHCGEDVPSHMDDECDLAGEIMQCEVKYQKAMGMA

>AlinOBP3

MDIRFGFIIACLAILSVANAISKEYSARMIAAKEKCQKEFNVTDSVVEDFMKRNIKPESKSGKCMVHCIMEEMGMIDDHKINTEQVKLGNKEKWDDPALVELANQVADTCDQEVFTEGRCKCLVAVEYMMCLATHGDEVGLPHVDFEDSQDS

>AlinOBP4

MRIFVIFTAALTCVMAGELPEEMKEMAQGLHDSCVEETGVDNGLIAPCAKGNFADDAKLRCYFKCVFGNLGVISDEGELDAEAFGSILPDSMQELLPTIKSCGGTTGSDPCDLAMNFNKCLQKADPVNFLVI

>AlinOBP5

MVLKMNLLLVVLVMSQVFFSVTEAAMSQAQMKQAMKTVRNMCIPKSGVDKEALAKMVNGEFDESDQKLKCYLGCVLGMMQAVKNNKINLTMVRNQITKMLAPERGQRILAAFESCATVTGDDNCGLAFRFAKCIYDTDKEAFIVP

>AlinOBP6

MGFKFVKYRSYFFVLVIRIILCIQIKAKELTDEQKEQIFAEIKNCMESTKLTDEEFESIMAKKELPTSIEGKCFTKCLMEKMEYLEEGGKINVIAVQAGMEENMEKESEITKAKEVIQQCADSVPPEDSCEYAYGISQCMYNKMKEAGISGS

>AlinOBP7

MNRPLLLLTAVLTVGSGQQEDCKTAPAGWPRRPPQCCDLPFPLEGMKKEFGSCIRQIGNRQSSAVPTAQAVRDARLCIEECVYKGLGFMDEHKLNKDQLLEQLKKGIADKKDWTKPMEGAVKRCHETITKRETPQEAACQDSAHEFTHCAMRELFLNCPASEWNNNDECNLVKSRMQACPNIPPPPPPPPQGFRGQGPPPQ

>AlinOBP8

MDTHFGLLIASLAILHTANAVINKDYLEKVVTAKDKCLKEFNVDDSVVEDFIVKYNKPQSESGKCMVACFMEERGMMKDGKTITEQVMLDNQEKWIAATHVNMGKEVIDTCDKEVPNEENDKCDLAVDYMMCLVKRGDEAGLPKMDVAQLKH

>AlinOBP9

MMELWKWRLALIIFGLVSCIQQTEGSQRTKQQPKSKTKESVVGATRPRDAKATECVNKVNANEEESASFFRKEIPETEAGKCLLACYLEGKGLIVGGKISSSGAARVAARAYPNNRVKTGNVKHILSHCGTIAGRESNNCEMAYKLADCTTTLSDKFRL

>AlinOBP10

MFFNSVFLLVVCVSSYVTKGQELPPPGDVKNKTVVFKNSFLRSAKYCSSIYETSTLAIMALLMSEKSDDQNGKCFLNCMLQRYRLMSQDGSYNKDKFKPFLEYIPDSKFLQSIRGNLKNCISEKDPDPCEKASKFIKCFYTRARNKGEIGASKEVIPADGF

>AlinOBP11

MKTFVGLIFAVALVEFASAISKEYHDKAIEAKNTCAKLHNVDDETIMTYWKNHQLPEKEPETCIVICYLKEMKLVVDGKVDADAWKASNKEKWDDEKHVAAADEIVDKCSAEVPPTENECEWGLALTKCALKHGKEAGIPPPDMEHPKRR

>AlinOBP12

MTTKLRSIGLVFIVSISYAFAYQELLKETIKKCQNGRDVTDDEVEEFTKPLVPKNEEERCLVACVFKEYKVIIDGHFDPVNALNVAKVVYKDYPDKVERIKDVLDHCGEDIPTHNDNECDLAGDIMKCEVKYLNSVPKMTSLEFLAGSMAATAEP

>AlinOBP13

MNISTRMISLTMAYLAAALVSGHRALDGILPQANQDECREESNFRGELNDDVGRNVTQELKCFAACSLMKLGIMNEKDGTVNMTRLDELIASHTPGKDAADVFKTTVVEPCMKEVKKSTDYCEYSYQLIACGMSKVP

>SfurOBP1

MLLEVCRFSVFLLAFSATVYGRFSEEEKQLMNQVHTQCVTETGTSEDLVNKATNGDFAEDENLKCYVKCIWSTLTVMDDDGNFDVGVLEVMLPADMKDIVMKAMSACIGAGGGSPCEKAFAVTKCLYKEAPADFFLP

>SfurOBP2

MSTLLNFVFVFLVCLCSYSEASPALTEAQIEQVGKAMANMCISSSGVQRSLITKAMTGEIEDDRKLKCFFGCIMEAVQVTKNGKMQPEVLKRRANAMLPKTMREMILPTIDSCSHIENEDKCELAYSIVKCHFSVNGKNPFFFNF

>SfurOBP3

MPPVFRTAHDKFESCLEELSSIFPPPPPPNGHHGPPPPGGPGGHGPPPPPPPGGRRGPPPGFGGPPGHEPPIFACAHECLFNKTGLLENGKLNVEALKKKLEGELGDDEVWKNLLQSIVDKCMESKDPPSNDMCTSGSHELARCVLRDMFMNCPQEKWKESDDCSNMKMKLEKCPELVPPMAMRLSQPPMP

>SfurOBP4

MCCDLPLVYRGTPELFKACREELGFPDHKPPPPPPSSDGHGPHGHPQRGMCVAECLFNRTGLLENGKINKEALKKALDEYLKTDGAWKDVATTTLEICYDAQTRGDFKPDNEKFTSGSSEFLKCFTRNLFMDCIPEKWTDSEECKKMKEKIDKCPKMLPPALFNKRPH

>SfurOBP5

MLYVSYFVIVTAASSAVITQIMAADSNNPDMQTVFNNCREEASATEDDIKTFRAQQIPSTTTGKCMLACMFNHSGLMKEGKYDSEGALKLVGQVFAADPVKLGKAKTLINTCSDEVKNENDKCEIASKIADCTVKMTSQVGLS

>SfurOBP6

MKCQVFLASFVLVAVFELGYAGLTPEKLKEIKPLIDTCIKESKVEEETLGKLHNGHEIPSSQSGKCFIACMAEHMKLMKDGKFEPAMTMEFIDKMVQDKVKADEIKKAVDDCFKSVPDGDKCEMAASLATCMKEHHAELAGMN

>SfurOBP7

MEEEMLSIGRVQRDADQTQEVADEYFKCKHRNLKTCCGKINLMKNYGDKGKIYGKQCYEEVVSAFKTNSSSTADDDDSMMDMFSCEKVKMIKLKHICVHECIGKKTKILKEDGSLNAEEIKQYAREYMFNEEWSKELGERALDKCLTQSYNSVTKMLDEYEIKCNPTSVQFHHCLWKEIEMTCPESKVDLKAKCVRLRERLRKQQAAGM

>SfurOBP8

MERTHVLIIAFAFIPFLSSAMQADFAMMQFPMQGTGTPMIQSIAGELKYCMDVNAEQNSDGLEDYLPLLFNEELPTSLGQKCFLTCLFNRFGLLKDGFLDAQTAKTLVETFYKDKHDEKTMANIAINVCRVSAVPDILNPCEIGFSLKSCFVDSNKKGKELRGKN

>SfurOBP9

MNTFQKFILSGMVVLAGAMLITAEDTTIKIKNQQSPHKQQQVYCQAPPTAPERLERIIEQCQDDIKTALLQEALNVLTDTSPRDLVKKTRSKREVFSGEEKRIAGCLLQCVYRKVKAVNDQGMPTVPGLVRLYSEGVQDRNYYVATVQAVQQCVSASQHFRYYNPQVLKEDGYTCDLAYDMFNCVSDKIEAFCGRTP

>SfurOBP11

MLLEVCRFSVFLLALSATVYGRFSEEEKQLMNQVHTQCVTETGTSEDLVNKATNGDFAEDENLKCYVKCIWSTLTVMDDDGNFDVGVLEVMLPADMKDIVMKAMSACIGAGGGSPCEKAFAVTKCLYKEAPADFFLP

>NlugOBP1

MKSFIVCIAVSYLLVANIKADEATSSSDAESLITSTTLSPASNESDAARSAIKEQLAKLTESCKTSSQANSDDAKIIGTESVPKTEGEKCFLQCVYTGFGIVKNDQFSVEGARLLAQKRFGAFPEELEKANQLIETCSKEAVKKDSKDKCPMGFLIRQCFVKNGQKINFFPKA

>NlugOBP2

MKCQIVLAALALATICEVSYAGLTPDKLKELKPLIDTCIKQSKVEEDTLGKLHNGHEIPSSQSGKCFIACMAEHMKLMKDGKFEPEMTMEFIDKMVQDKDKAAEIKKSLGECIKSVPEGDKCEMAAGLATCMKDHHAELAGMN

>NlugOBP3

MKASAAITLVFLSLAVFHCSEAKLDKAKKEAAIKKCQAETQATDEDVMKVRKEHIVPDSEEGKCFIACGFNSYDMLKDNRINLEGVNAFFEKLYDEQDKRDIAIKAAASCAATETVSGLNECHYAAKYFACMQRHPDFAKMKDDFDI

>NlugOBP4

MERTSVLIVFTFIPFLSSVLGANFLMMQQSMQGTQMPMIQSIASELKFCMDVNAEQNSDGLNDYLPLLFNEELPSTLGQKCFLTCLFNRFGLLKDGFLDTKTAKNLVETFYADKHDEKTMANIAINVCHVAAVPDALNPCEIGFSLKSCFVDSNKKGKELRGKN

>NlugOBP5

MGIYTTNLIFTLLGSAVVSGVFIDGRNEYRLTRQAPPDDECRPPRPGPNEDGVCCDMPPVFRTAHDKFESCLEELSSIFPPPPPPPHGHHGPPPPPPGARGPPPPPPPGGRRGPPPGFGGPPGHEPPIFACAHECLFNKTGMLENGKLNVEALKKKLEDELGENEVWKNLVQSIVDKCMESKDAPSNEMCTSGSHELARCVLRDMFMNCPQEKWKESDDCSNMKMKLEKCPELVPPMAMRLPHPPMP

>NlugOBP6

MSTFHKFVISGMVVLAGALFVTAEDTTIKIKTPSPHKHQQVYCQAPPTAPERLERIIEQCQDDIKTALQEALNVLTDTSPRDLVKKTRSKREVFSGEEKRIAGCLLQCVYRKVKAVDDQGMPTVPGLVRLYSEGVQDRNYYVATVQAVQQCVSASQHFRYYNPQVLKEDGYTCDLAYDMFNCVSDKIEAFCGRTP

>NlugOBP7

MLLEVCRFSVFLIALFATVNGRFTEEEKQLMNQVHSQCISETGTSEDLVTKATTGDFADDDNLKCYVKCIWSTLTVMDDEGNFDVGVLEVMLPADMKDTVMKAMNACTGVGGATPCEKAFAMTKCLYKEAPSDFFLP

>NlugOBP8

MVTSALMQTATAACLLLVTAYAYDFSDPYFNEHLQSAMEEIMEEEMLSIGRVQRDADQGQEVADEYFKCKHRNLKTCCGKINLMKNYGDKGKIYGKQCYEEVVSAFKTNSSSTADDDDSMMDMFSCEKVKMIKLKHICVHECIGKKTKILKEDGTLNPEEIKQYAREYMFNEEWSKELGEKALDKCLSQTYNSVTKMLDEYEIKCNPSSVQFHHCLWKEIELTCPESKVDLKAKCVRLRERLRKQQAAGM

>NlugOBP9

MPPVFRTAHDKFESCLEELSSIFPPPPPPPHGHHGPPPPPPGARGPPPPPPPGGRRGPPPGFGGPPGHEPPIFACAHECLFNKTGMLENGKLNVEALKKKLEDELGENEVWKNLVQSIVDKCMESKDAPSNEMCTSGSHELARCVLRDMFMNCPQEKWKESDDCSNMKMKLEKCPELVPPMAMRLPHPPMP

>BtabBCSP1

MHLFSVVVLVCCLLVAVLSAPAEFYTSQFDNIDIESILKNEKLLDNYFNCLMDEGPCTLEGRTLKSLLPDALNTSCAKCTEKQKKIARRVMTFYLDKYPANSARIIKKYDPENKFKDGIEKALLGSR

>BtabBCSP2

MFKVLVVLCVLGAAFVYAAPAEDKYTDKYDNINVDDILGSKRLLKSYLTCLLDKSPCTPEGSELKRLLPDALKTACSKCTEKQKEGAARIVERVTAEYPTEWKELSAKWDPTGEYWAKYKPLVQEYLKASA

>BtabBCSP3

MQVLTLVVLVGCAATAVLSADTYTTQFDNIDLEAILKNEKLVDNYTKCLMDEGPCTNEGRTLKKLLPDALKTACAKCTEKQKTGARKVIKFYQTQHPEDFKKLQQKYDPEGKFKAEFEKALFGQTL

>BtabBCSP4

MRCACVLLVLVICVWGTSGQRVGEGDVSRLLTNRDYVNRQINCVLDKGSCDNIGRQLKQAIPEVLGRQCKSCSARQLDNARKVVNYIRSNYPGPWSQIEAKYGRAAF

>BtabBCSP5

MKSVCVFAALVVACYAAPPAGVDEKLLSKYDNFDVDRVLNNDRVLANYIKCLMDEGSCTNEGRDLKKSIPDVLAGGCDKCTEKQKMVTEKVIKHLINKRPKDWDRLSKKYDPQGQYKNKYADLYEKVQKEAAKESKEPSKPTKDTTKVTKDTKESAKAPKA

>BtabBCSP6

MNKIVLALFALCALFGFSSAAAATKESTKESTYTNKYDNIDLGKILTNDRLFLNYFKCLMDEHTCSPDGAELKKVLPDALSNKCAKCTERQRSGSEKVIRHLIDNKPEMWAKLEAKYDPKGTYRKTYKNEAEKLGIKV

>BtabBCSP7

MIRVTLLLVAVALVGFVAGAPAPLEQSDLEKFENMDLSSILSNKRLRTAYVNCMVDKGPCTADAAEFKKILPDLTETQCADCSAKFKELIKKSVSTFQKDYPEDWKTLMAHFDPDNKRAADLEKFMSS

>BtabBCSP8

MKCVVLLAIFSVLAVYFVESAHLGPNFGAGDISGHLKNKNYILKQLNCVLGKGACDNVGKQLKVAIPEVLNKNCKGCTSQQAANAKRLITFMKSNYPAEWSKIAAKYKK‘

>BtabBCSP9

MSKYVFVLCVVAALAAVVSAADDFYSDKYDNIDLDSILASKRLIRNYMNCFQGKSPCTPEGTYLNQVLPEALKTECAKCTEKQREGAVKAIKKLSAEYPEEWKEITDKLDPTGEQYAKFKARFP

>BtabBCSP10

MGTFRALLLVVVSVCVFNVLRATPVPDEEKYSDKYDDVDYKSILNSKRLLNNYVKCLLDEGPCTAEGKALRDQLPDILATECKKCTDKQKKGSLDILEILQTEHQDAWTVLAKRWDPEDKLTKPLMEKLKKETGQA

>BtabBCSP11

MVCSESIVRCLTYFQVISLVYRLASAQSNNRATYTTKYDYINVDAVMKNERILKMLVECMLERGRCTREGLELKAAVPDALATDCAKCSQMQRKHASRVIAYLITYKKEYWNALATKYDPDGSYRRKYGIPPQPQELSAGAAIQPSPNLIKPTKTVKKTVTTVNNTNNLFKAKKRMTREEKKEMVRRRFPQLHFMPNMWASNIQKIEKKSERAPSIARRNVRRKAHTPTRRHSTQRTAPKRGQETG

>BtabBCSP12

MNPTVFLVILGQLSFVFSAISDDEYRLETLCSSPALEHFDITPILKNDRLVSSYFKCFMDEGPCTNEGKMVKRIIPEIMRTQCRNCNPTMRRIVRTVMKHMFQTRPRDVDDFFLKYDPHEMYYDDLIEFMDEDNDY

>BtabBCSP13

MNSLVLCAFVGSFIVGTLAAPAETYTTEFDGIDIDSVLKNEKLLDAYAKCLLDEGPCTREGRTLKTLLPDALETTCAKCSPTQKEKAKKVITFYMEKYPENAQKIMKKYDPTGKYRKALEEAFLGSL

>BtabBCSP14

MMKYCALSAVLACVFVVAVGRAQENQKQVQVPVNEMLNNTRMREAYFKCMSDKGPCTPDAAELKKVLPEAMTKKCAACTDTQKKILSKILDYMMEKDKATYKEIQEKYDPKNEYTKMREEEIKKEKAEEKKKPAEKDAPKKS

>BtabBCSP15

MIYVQILSFLCLSVLLAEAMPAPQTTRATISDEALESALNDKRYLMRQLKCALGEGVCDPVGRRLKTFAPLVLRGACPQCSPTETRQIQKVLSHIQRHHPKEWSKIVKQFTS

>BtabBCSP16

MFRLLLVTSLVLLVTGLPQKGPASTPRKQSVEEALGKKPEELPKTMKEALKRMEAVDVEKVLNNDRILTNYLKCFLNKGPCTSEAKNVKKFIALLVESRCVECDPKQRKIIKKSMQVVKTKKPREYQELIKLYDPKGTQIAELEKFFASSK

>BtabBCSP17

MNFLSVVVLVCCLFAAVLSAPAEFYTSQFDNIDIESILNNEKLLDNYFKCLMDEGPCTLEGRTLKSLVPDALNTSCAKCTDKQKQIARRVITFYLDKYPANSARIIKKYDPENKFKDGIEKALLGSR

>BtabBCSP18

MNLLSVVVLVCCLFAAVLSAPAEFYTSQFDNIDIESILNNEKLLDNYFKCLMDEGPCTLEGRTLKSLVPDALNTSCAKCTDKQKQIARRVITFYLDKYPANSARIIKKYDPENKFKDGIEKALLGSR

>BtabBCSP19

MHLLSVVVMVCCLVAAVLAAPAEFYTSQFDNIDIESILRNEKLLDNYFKCLMDEGPCTSEGRFLKNLLPDALNTKCAKCTDKQKKIARRVMTFYFDKYPANAARAIKKYDPENKLKDGLEKALLGAR

>MperCSP1

MNTLLLAVALCIAITMTVVQTAPAKYTTKYDNVNIDDILNNDRLVASYFKCLMETGKCTPEGEEIKRWLPEAIENKCENCSEKQKIGSEKIIKFLIEKKNDMWKQLEQKYDPQGLYKQRYSEEAKKLNLDV

>MperCSP4

MTNNNMNSPRCRPEIFSLLAVAAIATVLVHQPSTVHCADAGVYPPQQQQQEATMFTAPSGYYVSTYDHMDVGRLLRNNKVVAGFVKCFTNEGPCTPEGRLAKAYLLPEIIRTVCGKCTPRQKDMARLVIRHIYTYRRGDFDKIMQIYDTDGKKNEIIDFMNQK

>MperCSP5

MNCKVLIALCCVAVYAAHASPAGAATAAAASADEEIKDFPAYMKRFDKLNVEQVLNNDRVLASHLKCFLNEGPCVQQSRDLKRVIPVIANNGCNGCTERQMTTIKKSLNFLRTKKPVEWARLVKIYDPSGTKLNKFLDA

>AgosCSP1

MNILTIFCYVTVMCDTQVKPAVSAQRLQSVNQNVTPTNDGRKTIRETSSYPTRYDYIDIEAVMNNERIIKILFNCVMSRGPCTREGLELKRIVPDAIQTECAKCNERQRKQAGKVLAHLLQYKPEYWKMLVQKFDPNNVYLRKYMADNDDDEKLSLQKLSNDTTKKKRNI

>AgosCSP2

MAHLNLFVVLIASLIYFTSAAEEKYTTKFDNFDVDKVLNNNRILTSYIKCLLDEGNCTNEGRELKRVLPDALKTDCSKCTDVQKDRSEKVIKFLIKNRSTDFDRLTAKYDPTGEYKKNLEKFEKERASAKPLKA

>AgosCSP4

MDSRIAVVCVVLAAFAVDQTVGAPQKDAVAASGPAYTTKYDHIDVDQVLASKRLVNSYVQCLLDKKPCTPEGAELRKILPDALKTQCAKCNATQKNAALKVVDRLQKDYDAEWKQLLDKWDPKREHFQKFQQFLAEEKKKGFTKF

>AgosCSP5

MHCKVLIALCCVAVYAVQASPAGTATAAAVSADDEIKDFPAYMKRFDKLNVEQVLNNDRVLASHLKCFLNEGPCVQQSRDLKRVIPVIANNGCNGCTERQMTTIKKSLNFLRTKKPTEWARLVKIYDPSGTKLNKFLDA

>AgosCSP6

MIKLILAIAFCVSITMTVVQTAPAKYTTKYDNVNIDEILNNDRLVASYFKCLMETGKCTPEGEEIKRWLPEAIENKCEDCSEKQKLGSEKIIKFLFEKKNDMWKQLEAKYDPQGTYRQRYAEEAKKLNINV

>AgosCSP7

MSRSSSSVTMKVFVIAICVCAALARPEDVKVENKPAVIKSETLAAPLPTNIVKRATDTIQLDSSLPNVSEDVLDKALSDRRFVQRQLKCATGEGPCDPIGRKIKAHAPLVLRGMCVKCSQSEIKQIQRVMSHIQKNYPKEYTKMLKQYQSGF

>AgosCSP8 MNNIIMNNSRGRYGIFSLLAVTIAAIMLVHQPATVRCADGGIITPQQQQQQTMMFTAPTGYYVSTYDHIDVGRLLRNNKVVSGYVKCFVNEGPCTPDGKLVKAYLLPEIIRTVCGKCTPRQKDMARMVLKHIYTYRQADFEKIMQIYDTDGKRNEILAFMNH

>AgosCSP9

MSAFCLNSFILMTMITVIVTHATFTRSTKFDDRTGIDIHLVKRDTDDVNDDENSVESDEGFFYRFTHFFQDSSDKEDDDDDEKKPDFITTFDIFKLLDEEYAMQQFYCVINEDPCDEVGMRLKATIPEEINRNCERCTSTERNNIRRILNYVKKHYPQFWKRVEPIYKKKI

>AgosCSP10

MINTRPRKLVRCIRGVSISVAKGDDAVNAENKDDDSHLVNREEIQRYMSMMEKINIDQMLNNTRLMSNNVKCFLNEGPCTAHLREMKKMVPMLVKDSCSSCTKEQKIMMKKAMDAVKARRPNDYEKLSKFFDPEGKYEKKFLENLNESK

>AlucCSP1

MLKVLVLLAAVVCCVSAAATYTSKYDNIDLDEILSNTRLYKKYFDCLANKGKCTPDGKELKESLPDALKTNCAKCTKKQQEGTDKVLRHVLKNKPNDYKVLESIYDPTGIYRKKYEIEAEKRGIKLPGSH

>AlucCSP2

MASKLSVVLLIGAVGMVLAADKYTDKYDNIDLDEILGNQRLYQKYFDCIQGKGKCTPDGAELKETIPEALKTECAKCSDKQKAGVEKVLRHLIREKPDDYKVLEDQFDPEGVYRKKYEDLKKKVEEGKPIEY

>AlucCSP3

MKVAVLVLLCVGAALSAEVYTSKYDNIDVDKILSNDRILTQYIKCLMEEGNCTNEGKELKKTLPDALASGCTKCSEKQKAQTEKVLRHLSKNRPRDWNRLKNKYDPKGEYSKKYEKEAKAISA

>AlucCSP4

MKFVAALFVASVAVLAVEAADQYTTKYDNIDLDDILKNQRLYKKYFECLTNKGKCTPDGKELKEHLPDALKTGCSKCSEKQRAGSEKVIKHLLKNKPSDYAILEKIYDPQGSYKKKYEAEAKKLGINV

>AlucCSP5

MVGKLSVVLLIGAVGMVLAAELYTDKYDNIDVDEILGNQRLYQKYFDCIQGKGKCTPDGAELKKNIPEALQTDCAKCSEKQKAGVEKVLRHLINEKPEDYKVLEEQFDPEGVYRKKYEHLKKKVEEGKPV

>AlucCSP6

MVSKLSIVLLIGALADVWASELYTDKYDNIDVDEILGNQRLYQKYFDCIQGKGKCTPDGAELKKNIPEALQTDCAKCSEKQKAGVEKVLRHLINEKPEDYKVLEEQFDPEGVYRKKYEHLKKKVEEGKPIEY

>AlucCSP7

MVSKLSIVLLLGALADVWAAELYTDKYDNIDIDEILNNDRMYKNYFNCVMGNGKCTPDGLELKAKIPEALQTECAKCSDKQKKGAEKVLRFIINQKKDDYKLLEEKFDPEGVYRKKYEAQKKLAEEGKPIEY

>AlucCSP8

MLKVLVLLANAASTYTTKYDNIDLDEILSNQRLYKKYYDCLANKGKCTPDGKELKEALPDALKTNCSKCSKKQQEGTDKVLRYVLKNKPNDYKVLENIYDPSGNYRKRYEDEASKRGIKLPGSH

>AlinCSP1

MLKVLVLLAAVVCCVSAAATYTSKYDNIDLDEILSNTRLYKKYFDCLANKGKCTPDGKELKESLPDALKTNCAKCTKKQQEGTDKVFRHVLKNKPNDYKVLESIYDPPGIYRKKYEAEAEKRGIKLPGSH

>AlinCSP3

MISKLSMVLLIGAFADVWAAEQYTDKYDNIDIDEILNNDRMYKNYFHCVMGNGKCTPDGLELKAKIPEALQTECAKCTDKQKKEVEKVLRFIINQKKDDYKLLEEKFDPEGVYRKKYEAQKKLVEEGKPIEY

>AlinCSP2

MKVAVLVLLCVGAALSAEVYTSKYDNIDVDKILSNDRILTRYIKCLMEEGNCTNEGKELKKTLPDALASGCTKCSEKQKAQTEKVLRHLSKNRPRDWALLKTKYDPKGEYSKKYEKEAKALTA

>AlinCSP4

MRIILSAFLVAMACSLATCEMTEEEFYTKVFEEVDPDFILDNERILTSYLKCFYNEIECNAHAEVVKKSIPDVLATVCGRCSDKQKSIFKYSLNKFIPAHPKDWEKILSIYDPSGEAWPKVKAFIES

>AlinCSP5

MGHLTIVLLAAAFEVLTGSRAYTTHYDYIDVDQVLNNTRLYTKYVECLLGQGKCTPEARELRDKLPEALQTNCARCSERQASESHRVIRFLIQNRQEDFKLLEAKYDPSGLYFKRFEEETKRNVSLS

>AlinCSP6

MFYKLSVVVLMGILAGVWAADKYTDKYDNIDIDEILTNERLYKKYFDCIQGTGKCTPDGIELKEKIPEALKTECAKCNEKQKAGVEKVMRYLITKKPEDFKILEDKFDPEGVYRKKYEAQRKLVEEGKPVEY

>AlinCSP7

MNYKLSVILLIGVLASVWAASTYTDKYDNIDLDEILTNERLYKKYFDCIQGKGKCTPDGTELKEAIPDALKTECAKCNAKQKAGVEKVLRHLLTKKAEDYKILEDKFDPEGVYRKKYEAQKKLADEGKPIVL

>AlinCSP8

MDYKLSVMLLMGVLACAWAADKYTDKYDNIDIDEILNNERLYKKYFDCILGNGKCTPDGTELKETIPDALKTACAKCNDKQKAGVEKVLRHLLTKKAEDYKILEAKFDPEGVYRKKYEAQKKLAEEGKPIAL

>SfurCSP1

MFNLLTLVVCLSTIAVQIQAAPEEAQYTTKYDKINLDEILNNDRLFKSYFGCLMGGKCTPDGQTLRDILPDALETACSKCSDTQKAGTEKVFKFMIEKKPSEFADLEKKYDPNGKYRARYEADAEKFGIKV

>SfurCSP2

MVLADTPTTSPKVETKAVESGKSSSKDEIPDQTFDRYINNERYMLMQYECLMGNKPCDHVGRKLKAAVPLVVRGLGCPKCSQREEDQMKRIVSHVQRSYPDKWQKLIKKYGN

>SfurCSP3

MQLLYALVFGCTLVMVSSDMPQSTYPTKYDDYNPDDILKNDRLFNQYFICLTKKKGCTTAGELLSAIIPDALATSCAKCSAKQKAIGEKVIRFLYFNKPDEFAEMSKIYDPEGKYLEMYIASGGLI

>SfurCSP4

MKCPLLSVSCLWISLLALSSSASAATKEKDPERKALYRLEYIDIEKVLDNNRMLTNFIRCFLRKGPCSPEARDFRKLLPKLAKTMCSDCSPRQRFIIKKVFKHLMEERPKEWELLMDRFDPQRKYAERLDTFMVDMTTPSTTTTTTSTTPSTPMSSTTQRIIEILRTSTEMSNESSP

>SfurCSP5

MSEILVTSLIFMLLAASELGLGQQQQTQKPQQQNVDNIEMSIYDKMFENMDVNSLLKNHRLVDSYLKCFLNEGSCTHIGHEVKMMIPEVIRSRCATCGENQMRALKAGLRLFIVRRPDDWKRFLDVYDPDRTEWPHIKAFMESDD

>SfurCSP6

MKLALFCCLLGLVAAVSAQSEKSEKPEKYTTKYDYINVDEILSNDRLFNSYYKCLMGGKCTPGGPELRTHLPDALQTNCSKCSEKQKEFSDKVIKHLMDNKPEEFSALVKKYDPEGIYKDAFKPKHNQ

>SfurCSP7

MRASKASSLVAVLLIAVWGFTGVQAQQKSKDTRYTTRFDSIDVEVILKNERIFKRYMDCLLDKGRCTPEARELKRLLPEALKTECLKCSEVQRRQGAKVMGFIIKNKRPYWDLLLAKYDPQGIFRAKYNYNENNIEGVLKQLEREQQGLYGTYSNTTNTTNTVNSTSTRK

>SfurCSP8

MLKFTLTLLVLAVVSVNCGKLYKDRYTTKFDKIDLDEALNNQRLFESYLKCLMGDKCSPDGYELREALPDALATACAKCSDAQKAGTEKVIRFLIEKRPKEYALLEKEYDPEGIYRDKYKPIAQEKGIKI

>SfurCSP9

MRCLLLVAVVFAAFIAAARADEANKYTSKYDNIDIDKILKNDRVLSQYIKCLMGEGSCTQEGRELKRLLPDAIQSNCSKCSEKQRQASVKVMRHLRQSKERDWNRLLDKYDPQGDKRKNLKLD

>NlugCSP1

MFKNVLLVCLLVAVVSAKPKPAEKKQYTTKYDNIDLDEILNNQRLFDNYYKCLLGGKCTPDGQELREALPDALATACSKCTEKQRVGTEKVIKYLIEKKPTEYSELEKKYDPQGNYKRKYQAEAAKRGIKV

>NlugCSP2

MSKLPVTLVLMLAVFSVDCGKLYKDRYTTKFDKIDLDEALNNQRLFESYLKCLMGDKCSPDGYELREALPDALATACAKCSEAQKAGTEKVIRFLIEKRPKEYALLEKKYDPEGIYRDKYKPIAEMKGIKLD

>NlugCSP3

MKFLCVTIFECALIVVAFGMPQDTTYPTTYDDVNVDDILHNDRLFNRYFTCLTKKEGCTPEGKLLAATIPDALATTCAKCSAKQKTAAEKVIKYLYFNKRDKFDELAKIYDPESNYLNKYLVDGFPAKV

>NlugCSP4

MFLIAVWALSPRRLPWGLPWGGLAGVAAQQQAKNTRYTTRFDSIDVEVILKNERIFRRYMDCLLDKGRCTPEARELKRLLPEALKTECLKCSEVQRRQGAKVMAFIIKNKRPSWELLLAKYDPQGIFRAKYMYNENNIEAVLKQLEREQQGIYGTYSSTNSTTSSNSTSIR

>NlugCSP5

MRCLLLVAVVCAALVAVCHAQDSKYTSKYDNIDIDKILKNDRVLSQYIKCLMGEGSCTQEGRELKRLLPDAIQSNCSKCSEKQRSASVKVMRHLRQSRERDWNRLLDKYDPQGDKRKNLKLD

>NlugCSP6

MLWAARFIVLPLLFCVLQVWSAPADEKYTDIDFDSILANRRVLSSYVKCLTDKGPCTPQGKELKKIVPEVIQTSCTKCSPQQKKVVRNVITTMQSKYKDQWDLVVNKYDPKKQRSGELKAFLSGTD

>NlugCSP7

MASASSGTTSTTSAPKTAESASAKSSSKDEIPDQTFDRYINNERYMLMQYECLMGNKPCDHVGRKLKAAVPLVVRGLGCPKCSQREEDQMKRIVSHVQRSYPDKWQKLIKKYGN

>NlugCSP8

MSSTMLVFVAVLCFSAVLAKPADKYTTKYDNIDLDEVLSNQRLFDSYFKCLMGGKCTPDGQELRDALPDALATACEKCSEKQKEGTEKVMKFLIEKKPTEFAELEKKYDPQGTYRQKYKAEADKRGYSV

>NlugCSP9

MKSQQLLVSCLFICTWLVVLMAPSANAAPKEKDPERKALYRLEYIDIEKVLDNNRMLTNFIRCFLRQGPCTPEARDFRKLLPKLAKTMCSDCTARQRYIIKKVFKHLMEERPKEWELLMDRFDPQRKYAERLDTFMVDMTTRAPVTSSPMPSSPVTLTSSSVTMSSTTQRVIEILRTSTDMSNESRPAS

>NlugCSP10

MFMLLACSELGSGQQQQNVDNIEMSIYDKMFENMDVNSLLKNHRLVDSYLKCFLNEGSCTHIGHEVKMMIPEVIKSRCGTCGENQMRALKAGLRLFIVLRPDDWQRFLDVYDPDRKEWPHIKAFMDSDD

>NlugCSP11

MKSIILLVFVSMSAMVYRCRADEPSYPTSWDNVNIDEVLGNERLVQNYAKCLLEKGSCSPEGTELKKAIPDALKTGCTKCSDKQKAGAQKVIKWLVQKKPELWKEVVDKYDPSGEYTKKYEKEYQI
